# Measuring Change Over Time: A Systematic Review of Evaluative Measures of Cognitive Functioning in Traumatic Brain Injury

**DOI:** 10.3389/fneur.2019.00353

**Published:** 2019-05-08

**Authors:** Andrea D'Souza, Shirin Mollayeva, Nicole Pacheco, Fiza Javed, Angela Colantonio, Tatyana Mollayeva

**Affiliations:** ^1^Rehabilitation Sciences Institute, University of Toronto, Toronto, ON, Canada; ^2^Toronto Rehabilitation Institute-University Health Network, Toronto, ON, Canada; ^3^Acquired Brain Injury Research Lab, University of Toronto, Toronto, ON, Canada; ^4^Faculty of Life Sciences, McMaster University, Hamilton, ON, Canada; ^5^Department of Biology, University of Toronto Mississauga, Mississauga, ON, Canada

**Keywords:** measurements, neuropsychological tests, psychometrics, clinimetrics, systematic review

## Abstract

**Objectives:** The purpose of evaluative instruments is to measure the magnitude of change in a construct of interest over time. The measurement properties of these instruments, as they relate to the instrument's ability to fulfill its purpose, determine the degree of certainty with which the results yielded can be viewed. This work systematically reviews all instruments that have been used to evaluate cognitive functioning in persons with traumatic brain injury (TBI), and critically assesses their evaluative measurement properties: construct validity, test-retest reliability, and responsiveness.

**Data Sources:** MEDLINE, Central, EMBASE, Scopus, PsycINFO were searched from inception to December 2016 to identify longitudinal studies focused on cognitive evaluation of persons with TBI, from which instruments used for measuring cognitive functioning were abstracted. MEDLINE, instrument manuals, and citations of articles identified in the primary search were then screened for studies on measurement properties of instruments utilized at least twice within the longitudinal studies.

**Study Selection:** All English-language, peer-reviewed studies of longitudinal design that measured cognition in adults with a TBI diagnosis over any period of time, identified in the primary search, were used to identify instruments. A secondary search was carried out to identify all studies that assessed the evaluative measurement properties of the instruments abstracted in the primary search.

**Data Extraction:** Data on psychometric properties, cognitive domains covered and clinical utility were extracted for all instruments.

**Results:** In total, 38 longitudinal studies from the primary search, utilizing 15 instruments, met inclusion and quality criteria. Following review of studies identified in the secondary search, it was determined that none of the instruments utilized had been assessed for all the relevant measurement properties in the TBI population. The most frequently assessed property was construct validity.

**Conclusions:** There is insufficient evidence for the validity and reliability of instruments measuring cognitive functioning, longitudinally, in persons with TBI. Several instruments with well-defined construct validity in TBI samples warrant further assessment for test-retest reliability and responsiveness.

**Registration Number:** www.crd.york.ac.uk/PROSPERO/, identifier CRD42017055309.

## Introduction

Cognitive impairments are among the most important concerns for persons with traumatic brain injury (TBI). These impairments include a wide range of deficits in attention, memory, executive function, and behavioral and emotional difficulties, such as limited flexibility, impulsivity, reduced behavioral control, and inhibition, as well as other affective changes (1). Cognitive impairments directly impact their ability to maintain employment (2), personal and community independence (3), to participate in social activities (4), and their response to rehabilitation interventions (5). These are also among the main concerns of clinicians developing systems of care for TBI patients (6), and patients' family members and/or caregivers, who interact with and aid the injured persons on a daily basis (7). Cognition is a multi-dimensional construct, encompassing learning and memory, language, complex attention, executive functioning, perceptual-motor ability, and social cognition (8). Research to date has used numerous measures of cognitive functioning in persons with TBI longitudinally, to investigate its natural history (i.e., course over time) and the effectiveness of interventions aimed at improving cognition in clinical trials (9). The results were inconsistent, even when accounting for differences in time since injury and injury severity, with reports of improvement, decline, and no change over time (9). To elucidate the source of these inconsistencies, an important consideration involves investigation of the measures of cognitive functioning that have been utilized in the TBI population to date, to assess their suitability to perform the function for which they are intended. This route is one that has received relatively little attention in the discussion of generalization and interpretation of results of studies and this is a tremendous limitation, as selection of a measure affects the validity of the results reported (10). It has been argued that the usefulness of an outcome study or a clinical trial, in terms of the contribution made to the understanding of an issue and the potential to inform how the issue is viewed and treated in a clinical setting, hinges on the appropriateness of the measure used, and cannot be made up for even with otherwise superior design and execution (10). Measures used to study change in a construct over time are termed “evaluative,” and their most relevant psychometric properties, according to criteria developed by Feinstein (11) and Kirshner and Guyatt (12), are (i) construct validity, (ii) test-retest reliability, and (iii) responsiveness (11–13).

Construct validity refers to an instrument's ability to measure the construct it is intended to measure in the population of interest (e.g., cognitive functioning in the TBI population) (11). Developing a tool for measuring cognitive functioning that has construct validity is challenging because there is no generally accepted reference or gold standard instrument that is known to accurately define and measure the multidimensional construct, against which all new instruments could be compared (*convergent validity*). *Divergent validity* is another subcategory of construct validity, and it involves assessment of the relatedness of constructs thought to be unrelated and thus expected to yield scores on their respective measures that are not positively correlated (11). Finally, within construct validity there is also *known-groups validity*, which refers to the application of an instrument to two groups known or hypothesized to differ in the construct measured (11, 12). For an instrument to have construct validity, at least two of the construct validity subcategories must be assessed—convergent or divergent validity, and known-groups validity (11, 12).

Test-retest reliability concerns the extent to which application of the same instrument yields the same results in repeated trials under the same conditions (11, 13). This psychometric property is important for quantifying the degree of variance attributed to true differences in the construct under study over time, rather than systematic changes that occur when a procedure is learned (13).

Responsiveness refers to an instrument's ability to detect small, clinically significant differences in a construct of interest over time (12). This property is emphasized for instruments used in clinical trials, where the responsiveness of an instrument is directly related to the observed magnitude of the change in person's score, which may or may not constitute a clinically important difference (12). Responsiveness is inversely proportional to between-person variability in individual changes in score over time (12). In the TBI population, as the baseline variability increases, a larger treatment effect is needed to demonstrate intervention efficacy.

Finally, it is important to consider the specifics of the TBI population in the development and use of an instrument for cognitive functioning. Traumatic brain injury can impact not only cognition, but also behavioral and emotional functioning, and concentration, and this is expected to reflect in the ease of comprehension, extent of completion and overall burden on both the test taker and the administrator (in explaining the procedure and assisting with comprehension and completion) associated with administration of an instrument.

To identify the most appropriate instrument(s) for measuring cognitive functioning in the TBI population, we undertook a systematic review of all instruments used for this purpose. The objectives were to: (i) describe each evaluative instrument's key measurement properties (i.e., construct validity, test-retest reliability, and responsiveness); (ii) classify instruments according to the cognitive domains they assess; and (iii) summarize information relevant to their clinical and research applications. The present work intends to inform researchers and clinicians on each instrument's utility as an evaluative measure of cognitive functioning in the TBI population, while identifying pitfalls and future directions for their utility.

## Methods

This systematic review is part of a larger study that focuses on central nervous system (CNS) trauma [TBI and spinal cord injury (SCI)] as a risk factor of cognitive decline over time. For more information, the reader is referred to the published protocol (14) and registry with the International Prospective Register of Systematic Reviews (PROSPERO) (registration number CRD42017055309) (15).

### Primary Search: Studies of Cognitive Functioning in TBI

A comprehensive search strategy was developed in collaboration with a medical information specialist (JB) at a large rehabilitation teaching hospital. All English language peer-reviewed studies published from onset to December 2016 with prospective or retrospective data collection and a longitudinal design, identified in six electronic databases (i.e., MEDLINE, Central, EMBASE, Scopus, PsycINFO, and supplemental PubMed), were considered eligible. The following medical subject headings in MEDLINE were used to identify publications of interest (i) TBI terms: exp “brain injuries” or “craniocerebral trauma” or exp “head Injuries, closed” or exp “skull fractures” or “mTBI^*^2.tw.” or “tbi^*^2.tw” or “concuss^*^.tw.” AND (ii) cognition terms: exp “cognition” or exp “cognition disorders” or “neurocognit^*^.tw,kw.” Or “executive function” or exp “arousal” or “attention^*^.tw,kw.” or “vigilan^*^.tw,kw.” or exp “dementia” AND (iii) evaluation terms: exp “cohort studies” or “longitudinal studies” or “follow-up studies” or “prospective studies” or “retrospective studies” or “controlled before-after studies/or interrupted time series analysis” or exp “clinical trials” or exp “clinical trials as topic.” The search terms were adapted for use in other bibliographic databases. The reader is referred to the published protocol (14) and the PROSPERO registry (15) for the full search strategy. Additional studies were identified through review of reference lists of included articles.

### Inclusion and Exclusion Criteria

Studies were included if they met the following criteria: (i) focused on longitudinal change in cognitive functioning in adults (i.e., ≥16 years) with an established clinical diagnosis of TBI based on accepted definitions [e.g., Glasgow Coma Scale (GCS) score, duration of loss of consciousness, and post traumatic amnesia, etc.], excluding self-report; (ii) reported cognitive functioning outcome data at baseline assessment and follow-up as a score on a standardized measurement instrument; and (iii) the work was published in English in a peer-reviewed journal. Studies were excluded if they: (i) evaluated cognitive functioning in children/adolescents; (ii) studied persons with minor head injury (cases before 1993) without providing assessment criteria; or (iii) reported results in letters to the editor, reviews without data, case/public reports, conference abstracts, articles with no primary data, or theses.

### Selection and Quality Assessment of Studies

In the first stage of screening, two reviewers (NP and AD, or SM and AD) assessed study titles and abstracts for potential agreement with the inclusion criteria. In the second stage, each reviewer individually assessed the full texts of studies selected in the first stage to determine whether they met the inclusion criteria. Discrepancies in article inclusion/exclusion were resolved by discussion with TM.

Previously developed standardized forms were used to assess study quality (16) and to synthesize results (17). Study quality was assessed using the Quality in Prognosis Studies (QUIPS) guidelines (18). Assessments were based on the presence of six potential sources of bias (i.e., participation, attrition, prognostic factors, outcome measures, consideration of and accounting for confounders, and data analyses). Each study was assigned an overall “risk of bias,” and those with the greatest risk were excluded. Studies of a retrospective nature were automatically excluded from a “low risk” rating, as recommended by the Scottish Intercollegiate Guidelines Network (SIGN) (19). Any discrepancies between the two reviewers in quality assessment were resolved in discussion among the research team followed by independent review by the research supervisor (TM).

### Secondary Search: Studies of Measurement Properties of Abstracted Instruments

Instruments used to evaluate cognitive functioning in studies that met inclusion and quality criteria were abstracted. In collaboration with a medical information specialist (JB), proposed MEDLINE search filters were used to identify studies reviewing the abstracted instruments' measurement properties in TBI samples. Supplementary File 1 provides the terms and outputs from searches for each measure. The reference lists of eligible articles, instrument manuals and Google Scholar were reviewed for other relevant publications. Studies where the primary objective was not the evaluation of measurement properties were excluded.

### Evidence-Based Assessment of Instruments Evaluating Cognitive Functioning

Criteria for evidence-based assessment proposed by Holmbeck et al. (20) were utilized, previously applied in a systematic review of measurement properties of sleep-related instruments in the TBI population (17). Instruments used in at least two of the studies identified in the primary search were given ratings of “well-established,” “approaching well-established,” or “promising,” based on the following criteria: (i) use in peer-reviewed studies by different research teams; (ii) availability of sufficient information for critical appraisal and replication; and (iii) demonstration of validity and reliability in the TBI population (20).

### Descriptive Aspects of Instruments of Cognitive Functioning

To assess research and clinical feasibility, in-depth descriptions were completed following a previously developed format for instruments in medical research (17). The following descriptors were abstracted from data sources and reported: (i) general: purpose, content, response options, recall period (ii) application: how to obtain, method of administration, scoring and interpretation, administrator and respondent burden, currently available translations; and (iii) critical appraisal as reported by the researchers who utilized the instrument in TBI and other samples: strengths, considerations, clinical, and research applicability (17).

### Categorization of Instruments of Cognitive Functioning by Content

Content validity refers to the degree to which an instrument's items adequately reflect the construct of interest. For a measure to be sensitive to certain or all aspect(s) of cognitive functioning in a person with TBI, it needs to feature representative items or tasks that are part of the construct of cognition, as understood by the instrument's developer. As such, each instrument was categorized according to the cognitive domain(s) it assesses, focusing on those listed in the Diagnostic and Statistical Manual of Mental Disorders-Fifth Edition (DSM-5) (8): (i) complex attention, (ii) executive functioning, (iii) learning and memory, (iv) language, (v) perceptual-motor ability, and (vi) social cognition (8). An additional domain, information processing and reaction time, was included, given its relevance to the TBI population (21, 22). Instruments were then classified, based on the number of domains they assess, as either “global” (all domains), “multi-domain” (two or more domains), or “domain-specific” (one domain).

## Results

### Literature Search and Quality Assessment

**Figure 1:**
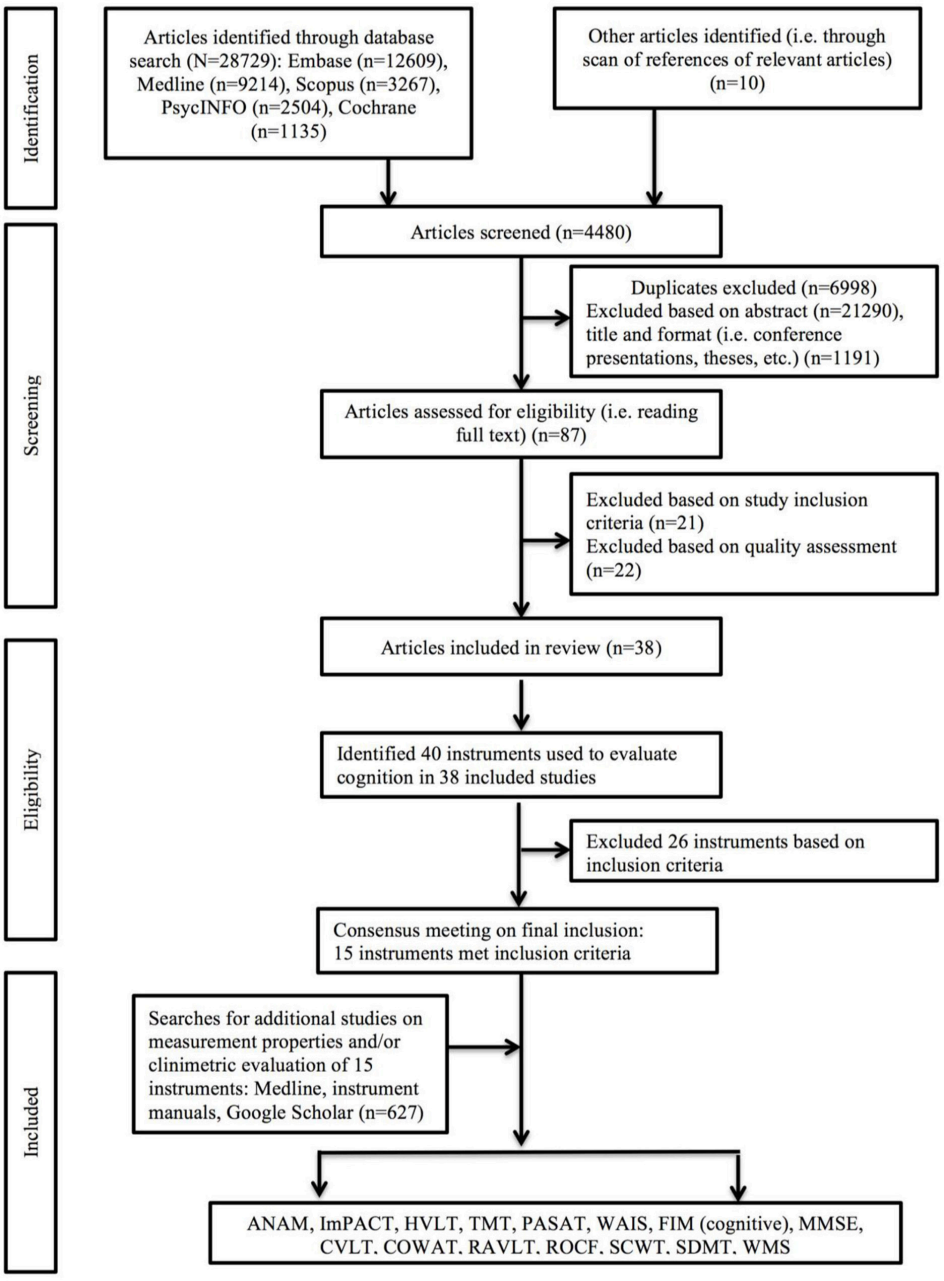
Flow chart documenting review procedure.

Of 29,566 studies identified in the primary search of articles assessing cognitive functioning longitudinally in the TBI population, 39 met inclusion and quality criteria (Figure 1): 14 studies involved patients from acute care (23–36), seven from the rehabilitation setting (37–43), ten involved college-age athletes (44–53), four were clinical trials (54–57), two involved samples from community care settings (58, 59), and another two involved military and veteran participants (60, 61). Sample sizes ranged from 10 (35, 55) to 509 (57), and consisted of mostly males (mean 76.6%, range 38-100%) with participant age ranging from 18 (57) to >60 years (57) (Table 1).

**Table 1:** Summary of study characteristics, including details on study sample, purpose, and instruments used to evaluate cognition.

**Authors**	**TBI sample size**	**TBI study setting**	**Injury severity (% of total), time since injury (at initial assessment)**	**Mean age/** **Mean age ± SD/** **Mean age (range)/** **Age group (%)**	**Sex** **(%M)**	**Study purpose**	**Instrument (s) used**
Bleiberg et al. (53), USA	64	Rehabilitation Institute/University Health Network	AAN: Grade I−9 (14.1); Grade II−49 (76.6); UKN−6 (9.4) TSI: 0–23 h	18.8 ± 0.7	100	To track cog impairment following sport con	**ANAM (CPT, MTS, MTH, Spatial Processing, SRT, Sternberg)**
Christensen et al. (37), Canada	75	Rehabilitation Institute	GCS: 6.97 ± 3.59 TSI: 1.5–2.5 m	37.37 ± 15.49	80	To examine patterns of cog rec in y following TBI	GPT; **RAVLT**; **Stroop; TMT A & B;** VF; **WAIS-III; WMS-III**
Covassin et al. (44), USA	79	College Sports Programs	AAN: GI−49 (62); GII-27 (34); GIII−3 (4) TSI: ≤3 d	NR	63.3	To identify any sex differences in post-con symps and cog function	**ImPACT**
Covassin et al. (45), USA	57	College Sports Programs	AAN (con–): GI-29 (80.6); GII-4 (11.1); GIII-3 (8.33)/(con+): GI-15 (71.4); GII-1 (4.76); GIII-5 (23.8) TSI: 1 d	Con−: 20.55 ± 1.54 Con+: 21.10 ± 1.69	47.2	To identify relationship b/w con history and post-con cog symps	**ImPACT**
Covassin et al. (46), USA	72 (college)	College Sports Programs	TSI: 2 d	M: 19.52 ± 1.08 F: 18.94 ± 1.55	NR	To identify any age and sex differences in symps, neurocog testing, and postural stability post-con	**ImPACT**
Chen et al. (54), China	15 (placebo)	Medical University Hospital	TSI: 1 d Last F/U: 12 weeks p/i	42.3 ± 14.05	66.67	To investigate effect of cerebrolysin therapy on cog recovery in mTBI	CASI, **MMSE**
Dikmen et al. (23), USA	421 overall: 130 (CT-) 133 (CT+, GCS 15) 158 (CT+, GCS 13-14)	Participants from 4 Prospective Longitudinal Investigations	GCS (CT–): 13–9 (7); 14–25 (19); 15–96 (74)/(CT+, GCS 15): 15–133(100)/ (CT+, GCS 13–14): 13–60 (38); 14–98 (62) TSI: 1 m	CT-: 28 ± 9.8 CT+, GCS 15: 35 ± 14.3 CT+, GCS 13-14: 38 ± 19	CT−: 71 CT+, GCS 15: 81 CT+, GCS 13-14: 73	To determine effect of com and uncom mTBI on outcome wrt controls	Finger Tapping; SRCL **TMT A & B; WAIS (DST, PIQ, Seashore Rhythm Test, VIQ)**
Failla et al. (58), USA	108	Level 1 Trauma Center	GCS: 8.02 ± 3.083 TSI: 6 m	34.19 ± 13.75	81.5	To determine if post-TBI cog rec is related to dopamine D2 receptor, and ankyrin repeat and kinase domain genes	**COWAT; CVLT-II;** D-KEFS (VF); **FIM-Cog; ROCF; Stroop; TMT A & B; WAIS-R (DG)**
Farbota et al. (24), USA	17	Level 1 Trauma Center	GCS−7.2 TSI– <3 m	34.5 ± 12.0	82.4	To examine brain volume loss in TBI patients using TBM and cog testing	**COWAT; TMT A & B**; **WAIS-III (DG);** WRAT-III (Reading)
Field et al. (47), USA	35 (college)	College Sports Programs	AAN: GI/II−23 (66); GIII−12 (34) TSI: ≤24 h	19.9 (17–25)	96	To evaluate patterns of rec wrt post-con symps and cognition	**HVLT**
Kersel et al. (25), New Zealand	65	Intensive Care Unit	GOS (6 m): Good−26 (40); mod−19 (29); sev−20 (31)/(1 y): Good−19 (29); mod−13 (20); sev−33 (51) TSI: 6 m	28 ± 11	75	To describe sev TBI effects wrt deficits and patterns of rec	AVLT; **COWAT**; **WAIS-R (BDT, DG, DST, FSIQ, Sim)**
Kontos et al. (60), USA	80	Army Medical Center	GCS: 15–80 (100) TSI: 1–7 d	Blast mTBI+: 31.05 ± 7.07 Blast mTBI-: 27.54 ± 5.57	100	To determine mTBI effect on cog performance and PTS symps in veterans w/ or w/o blast mTBI history	**ImPACT**
Kwok et al. (26), China	31	Hospital/ District Hospital	GCS: 13–15–31 (100) TSI: ≤1 w	38.60 ± 12.35	80.6	To examine changes in cog functioning of mTBI patients over a 3m period	AVLT; BVRT; DVT; FF; **SDMT**; VF; **WAIS (DG)**
Lee et al. (55), Korea	10 (placebo)	Hospital Trauma Center	CT: Ab−7 (70) TSI: 30.0 ± 6.5 d	35.5 ± 7.2	80	To compare effects of methylphenidate, sertraline and placebo for TBI neuropsych sequelae	CFFT; CRT (MRT, RRT, TRT); CTT; MAT; **MMSE;** STM; **WAIS (DST)**
Liberman et al. (27), USA	80 overall: 62 (APOE e4-) 18 (APOE e4+)	Shock trauma Center	GCS: 9–12–8 (8.0); 13–14–40 (50.0); 15–32 (40.0) LOC: #-64 (80.0) RGA: #-50 (62.5) TSI: 3 w	<30: 31.2% 30-49: 35.0% ≥50: 33.8%	60.0	To determine if short-term mTBI rec variability is related to *APOE* genotype	CRT; Dual Attention; GPT; Number Vigilance; **PASAT;** SRT1; **Stroop;** Word Recall; Word Recognition; Picture Presentation; Memory Scanning
Losoi et al. (28), Finland	74	University Hospital	ISS−3.9 ± 3.2 CT: Ab−7 (9.5) MRI: Ab−15 (20.3) LOC: #−27 (36.5); dur−0.9 ± 2.2 min PTA: #−68 (92.0) dur−2.6 ± 3.4 h TSI: 1 m	37.0 ± 11.8	60.8	To characterize mTBI rec	Finger Tapping; **RAVLT; Stroop; TMT A & B;** VF; **WAIS-III (DG, DST, SS)**
Macciocchi et al. (48), USA	24	College Sports Programs	AAN: GI−24 (100) LOC:<30 min−24 (100) TSI: 24 h	1 con: 19.5 2 cons:19.1	NR	To identify cog and behavioral effects of 1 vs. 2 cons	**PASAT; SDMT; TMT A & B**
Maksymiuk et al. (29), Poland	17	Air Force Institute of Aviation Medicine	GCS: 14–5 (29.4); 15–12 (70.6) LOC: Several s−7 (41.2); 1–20 min−6 (35.3); 30–60 min−5 (29.4) RGA: #−17 (100) PTA: #−17 (100)	22.1 (19-25)	100	To estimate rCBF and compare neuropsych results post-mTBI	Couve; **WAIS-R**
Mandleberg et al. (30), Scotland	149 overall: 51 (cohort 1) 98 (cohort 2)	Institute of Neurological Sciences	PTA: cohort 1–6 w (2 d-12 m); cohort 2–5 w (4 d-6 m) TSI: 3 m	1: 28.96 ± 13.14 2: 34.75 ± 15.11	1: 92.2 2: 87.8	To analyze relationship b/w PTA dur and cog functioning over time	**WAIS (PIQ, VIQ)**
McCrea et al. (49), USA	94	College Sports Programs	AAN: GI/II– 88 (93.2) LOC: #−6 (6.4); dur−30 s PTA: #−18 (19.1); dur−90 min RGA: #−7 (7.4); dur−120 min TSI: immediately	20.04 ± 1.36	NR	To characterize rate of impairment and rec following con	**COWAT** **HVLT;** SAC**; SDMT; Stroop** **TMT B**
Meier et al. (50), USA	17 (T1) 15 (T2) 13 (T3)	College Sports Programs	PTA: <1 min– 2 (11.8); 10–20 min−2 (11.8) RGA: <5 min−1 (5.9); 10–20 min−1 (5.9) LOC: #−0 (0) TSI: 1 d	20.57 ± 1.20	100	To characterize CBF rec and compare time of rec w/ cog and behavioral post-con symps	**ANAM (CDD, CDS, G/NG, memory search, MTH, MTS, procedural RT, spatial processing, SRT1 & 2)**
Ponsford et al. (31), Australia	123	Emergency & Trauma Center	GCS:13–15 −123 (100) LOC: #−111 (92.5; of 120 w/ known status); dur−61.44 ± 110 s PTA: #−118 (9.7); dur−103 ± 191 min TSI: ≤48 h	34.98 ± 13.13	74	To examine post-con symps, and cog, psych, and functional outcomes w/i uncom mTBI patients	**ImPACT**
Powell et al. (32), UK	35 (follow-up)	Hospital	GCS: 13–14 −5(14); 15 −27(77); UKN−3 (9) PTA: <1 h−15 (43); 1–24 h−11 (31) LOC: yes−19 (54); UKN−6 (17) TSI– ≤48 h	34.5	66	To assess MHI patients at admission and 3m	AMIPB; SOMC; **TMT B; WAIS (DG, DG Backwards)**
Prigatano et al. (38), USA	17 (control)	Neuropsychological Rehabilitation Program	AIR−1.82 (of n = 10) TSI:15.9 ± 13.6 m	23.5 ± 5.1	88.2	To evaluate effectiveness of neuropsych rehab by comparing patients to untreated controls	**TMT A & B; WAIS (BDT, DST, PIQ, VIQ, Vocab); WMS (Memory Quotient, LM, VR)**
Register-Mihalik et al. (51), USA	132	College Sports Programs	TSI–pre-season and 5 d postinjury	18.59 ± 1.09	65.2	To determine reliable change parameters for con measures w/i healthy controls and apply to con athletes	**ANAM (CDS, MTH, MTS, PRT, SRT 1 & 2, Sternberg)**
Roberston and Schmitter-Edgecombe (39), USA	49	Rehabilitation Program	GCS: 4.472 ± 8.400 PTA: 13.945 ±18.268 d TSI: 45.00 ± 35.14 d	37.796 ± 18.294	73.5	To examine change in self-awareness over course of TBI rec and relate to community re-integration	**COWAT; RAVLT; SDMT; TMT A & B; WAIS (LNS)**
Sandhaug et al. (40), Norway	41 overall 15 (mod) 26 (sev)	Rehabilitation Clinic University Hospital	T3 severity: mod−15 (36.6); sev−26 (63.4) TSI: 3 m	T1: 41 ± 18	T1: 77 T2: 75	To describe functional level ≤24m post-TBI and evaluate pre-injury/injury-related predictors	**FIM-Cog**
Schmitter-Edgecombe and Robertson (41), USA	21	Rehabilitation Program	GCS: 8.05 ± 4.50 PTA: 18.81 ± 11.65 h TSI: 41.20 ± 19.85 d	33.57 ± 14.55	71	To observe recovery of visual search processes in individuals with mod-sev TBI	**COWAT;** Preattentive and Attentive Visual Search Tasks; **RAVLT; SDMT; TMT A & B; WAIS-III (LNS)**
Snow et al. (59), Australia	24	Community	PTA: ≥14 d−24 (100) TSI: 17.8 ± 4.2 w	26.2 ± 7.8	66.7	To describe pattern of rec, and association b/w discourse and injury severity, executive function/verbal memory abilities, and psychosocial handicap w/i sev TBI patients over 2 years	CDA-M; FAS; **RAVLT**; **TMT B**
Sosnoff et al. (52), USA	36	College Sports Programs	AAN: G1–8 (22.2); GII−24 (66.7); G3–4 (11.1) TSI–baseline and post-injury	21.21 ± 1.49	80.6	To analyze the impact of mTBI on the relationship b/w cognitive and motor function	CRI; **ImPACT**
Sours et al. (33), USA	41	Shock Trauma Center/ University Medical Center	CT: Ab−9 (22.0) TSI: 7.66 ± 2.36 d	43.68 ± 16.98	73.2	To examine relationship b/w ANAM and IH-FC 1m post-mTBI	**ANAM (CDS, MTH, MTS, PRT, SRT 1 &)**
Till et al. (42), Canada	33	Rehabilitation Institute/University Health Network	GCS: 6.48 ± 3.34 LOC: 38.09 ± 18.31 d TSI: 54.03 ± 17.10 d	35.36 ± 14.52	75.8	To assess long-term cog decline following mod to sev TBI	**COWAT;** GPT**; RAVLT; SDMT; TMT A & B; WAIS (BDT, DG Forwards & Backwards); WMS (LM I & II)**
Tofil and Clinchot (34), USA	24	Teaching Hospital	PTA: 6.4 w(2.5–13.5 w) TSI: 19.5 d, (1–7 d)	28.6 (19–57)	70.8	To assess rec of auto and cog functioning w/ FIM-Cog	**FIM-Cog**
Vanderploeg et al. (61), USA	105	Defense and Veterans' Brain Injury Center	PTA: >1 d−100 (95) LOC: >30 min−105 (100) TSI: 32.4 ± 12.8 d	25.2 ± 6.4	94.3	To determine course of rec of memory processes in 1st y post-TBI	**CVLT**
Wang et al. (56), China	20 (control)	Clinical Trials Registry Platform of World Health Organization	GCS: 6.92 ± 1.38 CT/MRI: Ab−17 (85) TSI: 5.86 ± 4.54 y	28.64 ± 10.13	75.00	To study effects of umbilical cord mesenchymal stem cell transplantation in TBI patients and compare to untreated controls	**FIM-Cog**
Whyte et al. (43), USA	108 overall: 72 (early rec) 36 (late rec)	National Institute on Disability and Rehabilitation Research	GCS: 25 %ile−3; 50 %ile−4; 75 %ile−6 LOS: 25 %ile acute/rehab−23/28 d; 50 %ile−32/45 d; 75 %ile−46/68 d TSI: 92.5 d	26.2 ± 15.5	68	To describe 5-year outcomes of TBI patients who could not follow commands at rehab admission	**FIM-Cog**
Wylie et al. (35), USA	18 overall: 8 (cog rec) 10 (no cog rec)	Tertiary Care Academic Medical Center	GCS: cog rec+-15; cog rec–−15 LOC: cog rec+-3 (38); cog rec—1 (13) CT (Ab): cog rec+-1 (13); cog rec—0 (0) TSI: cog rec+-2.2 ± 1.0 d; cog rec—2.0 ± 0.6 d	Cog rec+: 30.0 ± 15.6 Cog rec-: 26.2 ± 9.6	Cog rec+: 38 Cog rec-: 60	To understand early effect of con on working memory	**ImPACT**
Zafonte et al. (57), USA	509 (placebo)	8 Level 1 Trauma Centers	Initial n = 606 GCS: com mild−404 (66.7); mod/sev– 202 (33.3) Head AIS: ≤4–171 (28.5); >4–429 (71.5) PTA: ≤24 h−121 (25.8); >24 h−347 (74.2) TSI: 90 d	n = 606: 18-30: 199 (32.8) >30-45: 142 (23.4) >45-60: 184 (30.4) >60: 81 (13.4)	n = 606: 73.8	To determine effectiveness of citicoline on functional and cog status in com mild, mod and sev patients	**COWAT; CVLT;** GPT, **Stroop; TMT A & B; WAIS-III (DG, PSI)**
Zaninotto et al. (36), Brazil	40	USP Clinics Hospital	Severity: mod−16 (40.0); sev−24 (60.0) TSI: 6 m	28.7 ± 9.4	87.5	To evaluate visual memory performance post-TBI	GPT; **ROCF; WAIS-III**

*Cognitive instruments discussed within this review are bolded*.

*AAN, American Academy of Neurology; Ab, abnormal; AGCT, Army General Classification Test; AIR, Average Impairment Rating; AIS, Abbreviated Injury Score; AMIPB, Adult Memory and Information Processing Battery; ANAM, Automated Neuropsychological Assessment Metrics; APOE e4, Apolipoprotein E; assoc, association; auto, automatic; AVLT, Auditory Verbal Learning Test; b/w, between; BCT, Bell Cancellation Task; BDG, backward Digit Span; BDT, Block Design Test; BVRT, Benton Visual Retention Test; CASI, Cognitive Abilities Screening Instrument; CBF, cerebral blood flow; CFFT, Critical Flicker Fusion Threshold; CM, Color Matching; cog, cognitive/cognition; com, complicated; con, concussion; COWAT, Controlled Oral Word Association Test; CPT, Continuous Performance Task; CRI, Concussion Resolution Index; CRT, Choice Reaction Time; CT, computed tomography; CTT, Compensatory Tracking Task; CVLT, California Verbal Learning Test; d, days; DA, Dual Attention; DCT, Digit Cancellation Task; DG, Digit Span Test; diff, different/differences; D-KEFS, Delis-Kaplan Executive Function System; DM, Design memory; DST, Digit Symbol Test; dur, duration; DVT; Digit Vigilance Test; EMQ, Everyday Memory Questionnaire; exec func, executive function; F, female; FDG, forward Digit Span; FF, Figural Fluency; FIM-Cog, Functional Independence Measure-Cognitive Subscale; FSIQ, Full Scale Intelligence Quotient; FT, Finger Tapping; func, functional; G, Grade; GCS, Glasgow Coma Scale; GOS, Glasgow Outcome Scale; GPT, Grooved Pegboard Test; h, hours; HVLT, Hopkins Verbal Learning Test; IH-FC, inter-hemispheric functional connectivity; ImPACT, IMmediate Post-concussion Assessment and Cognitive Testing; IQ, Intelligence Quotient; ISS, Injury Severity Score; LM, Logical Memory; LOC, loss of consciousness; m, months; M, male; MAT, Mental Arithmetic Test; max, maximum; min, minutes; MHI, mild head injury; MIST, Memory for Intentions Screening Test; MMSE, Mini-Mental Status Examination; mod, moderate; MQ, Memory Quotient; MRI, magnetic resonance imaging; MS, Memory Scanning; mTBI, mild traumatic brain injury; MTH, Mathematical Processing; MTS, Matching to Sample; neurocog, neurocognitive; neuropsych, neuropschyological; NR, not reported; NV, Number Vigilance; P/A VST, Pre-attentive/Attentive Visual Search Tasks, PASAT, Paced Auditory Serial Addition Test; perf, performance; PIQ, Performance Intelligence Quotient; plac, placebo; PP, Picture Presentation; PRO, Procedural Reaction Time; psych, psychological; PTA, post-traumatic amnesia; PTS, post-traumatic stress; RAVLT, Rey Auditory Verbal Learning Test; rCBF, regional cerebral blood flow; rec, recovery; rehab, rehabilitation; ROCF, Rey-Oesterrieth Complex Figure Test; RGA, retrograde amnesia; SD, standard deviation; SDMT, Symbol Digit Modalities Test; sev, severe; Sim, Similarities; SM, Symbol Matching; S-NAB, Neuropsychological Assessment Battery – Screening Module; SOMC, Short Orientation Memory and Concentration Test; SPA, Spatial Processing; SRCL, Selective Reminding Test Sum of Recall; SRT, Simple Reaction Time; ST, Stroop Test; STN, Sternberg Memory Search; symp, symptoms; T, time; TAPI, Test d'Attention Partagée Informatisé; TBI, traumatic brain injury; TBM, tensor based morphometry; TLM, 3 Letter Memory; TMT, Trail Making Test (full version, or part A/B); TOMM, Test of Memory Malingering; TSI, time since injury; UKN, unknown; uncom, uncomplicated; VEM, Verbal memory; vets, veterans; VF, Verbal Fluency; VIQ, Verbal Intelligence Quotient; Voc, Vocabulary; VR, Visual Reproduction; w, week; w/, with; w/i, within; w/o, without; WAIS, Wechsler Adult Intelligence Scale; WMS, Wechsler Memory Scale; WRAT, Wide Range Achievement Test; wrt, with respect to; y, years*.

All 39 studies were assessed as having “Partly” or “No” on all bias criteria. Twenty-nine studies were of fair quality (23, 26, 29–32, 34, 37–45, 48–54, 54–61), ten were of good quality (24, 25, 27, 28, 33, 35, 36, 46, 47, 53) and none were of high quality. Studies were most frequently penalized by the SIGN criteria for unknown reliability and validity of the utilized instruments, incomplete statistical analysis, and selection bias due to study attrition (Supplementary File 2).

### Instruments Measuring Cognitive Functioning

Within the 39 studies, 15 instruments were used more than once. The Mini Mental State Examination (MMSE) (54, 55), Hopkins Verbal Learning Test (HVLT) (47, 49), Paced Auditory Serial Addition Test (PASAT) (27, 48), and Rey-Osterrieth Complex Figure Test (ROCF) (36, 58) were each used twice; the California Verbal Learning Test (CVLT) (57, 58, 61) and Wechsler Memory Scale (WMS) (37, 38, 42) were used three times; the Automated Neuropsychological Assessment Metrics (ANAM) (33, 50, 51, 53) and FIM-Cog (Functional Independence Measure-Cognitive Subscale) (34, 40, 43, 56, 58) were used four and five times, respectively; the Rey Auditory Verbal Learning Test (RAVLT) (28, 37, 39, 41, 42, 59), Stroop Color Word Test (SCWT) (27, 28, 37, 49, 57, 58), and Symbol Digit Modalities Test (SDMT) (26, 39, 41, 42, 48, 49) were each used six times; the Immediate Post-concussion Assessment and Cognitive Test (ImPACT) (31, 35, 44–46, 52, 60) and Controlled Oral Word Association Test (COWAT) (24, 25, 39, 41, 42, 49, 57, 58) were used seven and eight times, respectively. The most frequently used instruments were the Trail Making Test (TMT) (23, 24, 28, 32, 37–39, 41, 42, 48, 49, 57, 59) and the Wechsler Adult Intelligence Scale (WAIS) (23–26, 28–30, 32, 36–39, 41, 42, 55, 57, 58), used 13 and 17 times, respectively.

### Assessment of TBI

Diagnostic criteria and definitions of TBI varied considerably between studies included in this review (Table 1). Nineteen studies used a combinatorial approach to confirm and assess TBI. This included the use of such tools as the Glasgow coma scale (GCS), duration of posttraumatic amnesia (PTA) and/or loss of consciousness, neuroimaging results [i.e., magnetic resonance imaging (MRI), computed tomography (CT)], and clinical evaluations and tests (30, 31, 33, 34, 36, 40, 42–44, 46–49, 53, 61–65). Five studies used the American Academy of Neurology (AAN) graded concussion assessment test (44, 53, 55, 59, 66), six used GCS scores (38, 39, 50, 56, 58, 67), one study assessed CT scans (60), one—MRI scans (57), and one assessed PTA (37) alone to confirm TBI. Two studies used other methods, including description of damage and/or lesions based on medical records, and diagnoses of referring professionals (29, 68).

### Injury Severity in Samples Assessed

Three measures were used to assess cognitive functioning in mild TBI (mTBI) samples only (ANAM, HVLT, ImPACT), while the rest were applied to samples of varying injury severities. Among the most commonly used measures were the TMT, used in 11 studies, of which six (23, 28, 32, 42, 48, 49) comprised mTBI samples, three—mixed injury severity samples (24, 39, 41), and two—severe TBI samples (58, 59). The COWAT was used in eight studies, of which two (42, 49) featured mTBI samples, four (24, 39, 41, 57)—mixed injury severity samples, and two (25, 58)—severe TBI samples. Several versions of the WAIS were used seven times: once (23) in a study of mTBI participants, twice (36, 41) in samples of mixed injury severities, and four times (25, 30, 38, 58) in severe TBI samples (**Table 3**).

## Evaluation of Measurement Properties

### Construct Validity

Convergent, divergent, and/or known-groups validity (62) were reported for all instruments in TBI samples of all severities and mixed-severity samples (39, 53, 63–81). Where construct validity was evaluated, at baseline or follow-up assessments, correlation strength between scores of instruments measuring the same construct, or scores of groups of people with known differences in cognitive functioning, were not always in line with clinical expectations. There is evidence of moderate to strong convergent validity of the original version of CVLT in mixed severity TBI samples (64), FIM-Cog in severe and mixed TBI samples (67); ImPACT in mTBI samples (65, 66); MMSE in mixed severity TBI (80); PASAT in mild and mixed severity samples (69); ROCF in severe and mixed samples (70, 71); SCWT, and WAIS and WMS in all TBI severity samples (63, 72–75, 77, 78).

Divergent validity hypotheses were tested by analyzing the correlation of the PASAT with measures of intellectual, mathematical, and verbal abilities, academic achievement and complex motor skills (i.e., *r* = 0.29–0.59, *p* < 0.05), all of which were significant positive correlations (69). Correlations of the ImPACT with difficulty concentrating and remembering were negative (i.e., *r* = −0.48– (−0.41), *p* < 0.01) (Table 2) (65).

**Table 2:** Quality assessment of the 15 selected instruments based on criteria proposed by Holmbeck et al. (20).

**Measures**	**Frequency of use by different investigators**	**TBI severity**	**Frequency of use by same team**	**Details for critical evaluation**	**Test-retest reliability/** **responsiveness**	**Construct validity (concurrent, divergent/convergent, known-group)**
ANAM	**4x** (33, 50, 51, 53)	Mild TBI (33, 50, 51, 53)	N/A	Can be purchased at vistalifesciences.com	ANAM4: T1 and T2 357 ± 88 d (range 99–637) NA/ decline in CDS, CDD, M2S, MTH, PRT, SRT, and SRT2; 48% w decline on ≥2 subtests (68)	**Known Groups** • SPA/MTH: sd b/w TBI and HCs (*p* < 0.05) (53) • CDS, CDP, PRT, SRT, SRT2: sd b/w concussed and non-concussed (*p* < 0.05) (68)
COWAT	**8x** (24, 25, 39, 41, 42, 49, 57, 58)	Mild TBI (42, 49) Severe TBI (25, 58) Mixed TBI (24, 39, 41, 57)	**2x** (39, 41)	Can be purchased at parinc.com	U/K	**Known Groups** • sd b/w TBI and HCs at BS (*p* < 0.01) (72)
CVLT	**3x** (57, 58, 61)	Version II Severe TBI (58) Original version Mixed TBI (57, 61)	N/A	Can be purchased at pearsonclinical.com	U/K	**Convergent** • CVLT w/ RAVLT: 1and5, 1–5, B, SDR, % SDR, and Int (*r* = 0.49–0.83; *p* < 0.001) (64)
FIM-Cog	**5x** (34, 40, 43, 56, 58)	Severe TBI (34, 41, 56) Mixed TBI (43, 58)	N/A	Not available online; must contact UDSMR to subscribe	U/K	**Convergent** • FIM-Cog w/ DRS, FIM+FAM mot and cog, LCFS, PTA: *r* = 0.41–0.95; *p* < 0.05 (67)
HVLT	**2x** (47, 49)	Mild TBI (47, 49)	N/A	Can be purchased at parinc.com	• HVLT-R TR, DR, % Retained, discrim.: *r* = 0.64–0.82; *p* < 0.05 (83)	**Known Groups** NS b/w TBI and HCs in HVLT total, sd in HVLT delayed (*p* = 0.02) (79)
ImPACT	**7x** (31, 35, 44–46, 52, 60)	Mild TBI (31, 35, 44–46, 52, 60)	**4x** (44–46, 60)	Can be purchased at impacttest.com	U/K	**Divergent** • VEM w/ difficulty concentrating and remembering: *r* = −0.48 to −0.41; *p* < 0.01 (65) **Convergent** • RT w/ SDMT, verbal/visual memory, feeling foggy, difficulty remembering: *r* = 0.36–0.70, *p* < 0.05) (66)
PASAT	**2x** (27, 48)	Mild TBI (48) Mixed severity TBI (27)	N/A	Can be purchased at pasat.us	U/K	**Convergent** • 2.0s and measures of attention (*r* = 0.35–0.49, −0.35, *p* < 0.001) (69) **Divergent** • 2.0 s and measures of intellectual ability, mathematical knowledge, verbal ability, academic achievement, and complex mot skills (*r* = 0.29–0.59, *p* < 0.05) (69)
RAVLT	**6x** (28, 37, 39, 41, 42, 59)	Mild TBI (28), Mixed severity TBI (37, 39, 41) Severe TBI **s** (42, 59)	**2x** (37, 42) **2x** (39, 41)	Can be purchased at wpspublish.com	U/K	**Known Groups** • List learning/delayed: sd b/w TBI and HCs at BS (*p* < 0.01) (39)
ROCF	**2x** (36, 58)	Mixed severity TBI (36) Severe TBI (58)	N/A	Can be purchased at parinc.com	U/K	**Convergent/divergent** • Copy w/ GPT NDH: *r* = 0.37; *p* < 0.01 (70) • IR w/ SDMT, GPT NDH: *r* = 0.35–0.37; *p* < 0.01 (70) • RCFT rec. w/ COWAT: *r* = 0.35;*p* < 0.01 (70) • RCFT total w/ total voxel # on L/R thalamic RSNs: ρ = −0.55; *p* = 0.003 (71)
MMSE	**2x** (54, 55)	Mild TBI (54), Mixed severity TBI (55)	N/A		U/K	**Convergent Validity** • w/ MoCA score (*r* = 0.852, *p* < 0.001) (80) **Known Groups** • sd b/w TBI and stroke at (t = 3.13, *p* < 0.001, most difference in orientation domain) (81)
SCWT	**6x** (27, 28, 37, 49, 57, 58)	Mild TBI, (27, 28, 49) Mixed severity TBI, (37, 57, 58)	N/A	Can be purchased at parinc.com	U/K	**Convergent/divergent** • w/ informant CFQ: *r* = 0.47; *p* < 0.05 (72) • w/ SADI: *r* = 0.41; *p* < 0.01 (73) • CW w/ ADL, informant CFQ, QOLIBRI: *r* = 0.40–0.55, −0.40; *p* < 0.05 (74)
SDMT	**6x** (26, 39, 41, 42, 48, 49)	Mild TBI, (26, 48, 49) Mixed severity TBI (39, 41) Severe TBI (42)	**2x** (39, 41)	Can be purchased at wpspublish.com	U/K	**Known Groups** • Oral/Writ: sd b/w TBI and HCs at BS (*p* < 0.01) (72)
TMT	**14x** (23, 24, 28, 32, 37–39, 41, 42, 48, 49, 57, 59)	Mild TBI (23, 28, 32, 48, 49), Mixed severity TBI (37–39, 41, 58), Severe TBI (24, 42, 59)	**2x** (37, 42) **2x** (23, 57) **2x** (39, 41)	Can be purchased at www.mcssl.com	U/K	**Known Groups** • A/B: sd b/w TBI and HCs at BS (*p* < 0.01) (39) • B: sd b/w TBI and non-TBI (*p* = 0.047) (39)
WAIS	**17x** (23–26, 28–30, 32, 36–39, 41, 42, 55, 57, 58)	Mild TBI (23, 26, 28, 29, 32, 36, 55), Mixed severity TBI (37–39, 41, 57, 58) Severe TBI (24, 25, 30, 42)	**2x** (37, 42) **2x** (23, 57) **2x** (39, 41)	Can be purchased at pearsonclinical.com	U/K **Responsiveness** • Info, Comp, DST, PC, PA, PIQ, FSIQ: ↑0–3 to 7–12 m (*t* = 2.24–3.44; *p* ≤ 0.05) (84) • DST, BDT, PA, OA, PIQ, FSIQ: ↑ 4–6 to >13 m (*t* = 2.19–2.68; *p* ≤ 0.05) (84) • Info, Comp, Arith, DG, Vocab, DST, PC, BDT, PA, VIQ, PIQ, FSIQ: ↑ 0–3 to >13 m (*t* = 2.06–4.93; *p* ≤ 0.05) (84)	**Convergent** • WAIS Vocab w/ early MRI: *r* = −0.43; *p* < 0.05 (75) • WAIS Sim, Vocab, DST, BDT, OA w/ late MRI: *r* = −0.86 to −0.48; *p* < 0.01 (75) • WAIS-R BDT w/ VFD Total and Rotation: *r* = 0.47, −0.45; *p* < 0.05 (63) **Known Groups** • WAIS-R DG: sd b/w TBI and HCs (p ≤ 0.001) (76) • WAIS-III LNS: sd b/w TBI and HCs at BS (*p* < 0.01) (39) • WAIS-IV: sd b/w TBI and non-TBI (*p* < 0.01) (39) • WAIS-IV: mTBI positive vs negative combat-exposed military personnel (*p* < 0.17) (82)
WMS	**3x** (37, 38, 42)	Mild TBI (42) Mixed TBI (37) Severe TBI (77)	**2x** (37, 42)	Can be purchased at pearsonclinical.com	U/K	**Convergent** • WMS MQ/VM and L/R-brain: sig↓ pre- to post-operation (77) • WMSandWAIS FSIQ: *r* = 0.75–0.83 (77) • WMS-R VM and PTA: *r* = −0.53; *p* < 0.05 (78) • WMS-R VEM/VM/GM/Attention/ Concentration/DR and IMIS: *r* = −0.65 to −0.44; *p* < 0.05) (78)

*ADL, activities of daily living; ANAM, Automated Neuropsychological Assessment Metrics; Arith, Arithmetic; BDT, Block Design Test; BS, baseline; b/w, between; CFQ, Cognitive Failures Questionnaire; cog, cognitive/cognition; Comp, Comprehension; COWAT, Controlled Oral Word Association Test; CW, Color Word; d, days; DG, Digit Span; discrim., discriminability; DR, delayed recall; DRS, Disability Rating Scale; DST, Digit Symbol Test/Digit Symbol Coding/Coding; CVLT, California Verbal Learning Test; FAM, Functional Assessment Measure; FIM, FIM instrument, cognitive subscale (formerly Functional Independence Measure); Freq, frequency; FSIQ, Full Scale Intelligence Quotient; GM, General Memory; GPT, Grooved Pegboard Test; HCs, healthy controls; HVLT, Hopkins Verbal Learning Test; IMIS, Inpatient Memory Impairment Scale; ImPACT, Immediate Post-concussion Assessment and Cognitive Test; Info, Information; Int, Intrusions; ICC, intraclass correlation coefficient; IR, Immediate Recall; LCFS, Levels of Cognitive Functioning Scale; LNS, Letter Number Sequencing; L/R, left/right; MoCA, Montreal Cognitive Assessment; mot, motor; MRI, magnetic resonance imaging; MTH, Mathematical Processing; MQ, Memory Quotient; N/A, not applicable; U/K, unknown; NDH, non-dominant hand; OA, Object Assembly; PA, Picture Arrangement; PASAT, Paced Auditory Serial Addition Test; PC, Picture Completion; perf, performance; PIQ, Performance Intelligence Quotient; PTA, post-traumatic amnesia; QOLIBRI, Quality of Life after Brain Injury; RAVLT, Rey Auditory Verbal Learning Test; RCFT, Rey Complex Figure Test and Recognition Trial; ROCF, Rey-Osterrieth Complex Figure Test; RSNs, resting state networks; RT, Reaction Time; SADI, Self-Awareness of Deficits Interview; sd, significant difference; SDMT, Symbol Digit Modalities Test; SDR, short delay recall; sess, sessions; Sim, Similarities; SPA, Spatial Processing; TBI, traumatic brain injury; TMT, Trail Making Test; TR, total recall; UDSMR, Uniform Data System for Medical Rehabilitation; w/, with; WAIS (-R/III/IV), Wechsler Adult Intelligence Scale (-Revised/Third Edition/Fourth Edition); WMS (-R/III/IV), Wechsler Memory Scale (-Revised/Third Edition/Fourth Edition); Writ, written; VEM, Verbal Memory; VFD, Benton Visual Form Discrimination Test; VIQ, Verbal Intelligence Quotient; VM, Visual Memory; Vocab, Vocabulary. Bold values designate number of uses of the instrument, meant to distinguish from the citations beside these values*.

Known-groups validity was reported for several domains of the ANAM and HVLT in mTBI samples (53, 68, 79); COWAT in mixed and severe TBI samples (72); list learning/delayed recall from the RAVLT in all injury severity samples (39); oral and written SDMT in mild and mixed severity samples (72); MMSE in a TBI sample of unknown severity (81); TMT, WAIS-R and WAIS-III in mixed severity samples (39, 72, 76, 77), and WAIS-IV in mTBI sample (82). Significant differences were observed between the scores of persons with TBI and healthy controls, and TBI and other neurological populations (Table 2).

### Test-Retest Reliability

Test-retest reliability refers to the consistency of scores attained by the same patient over the course of several attempts at different times (62). It concerns the stability of the instrument's performance over a period of time, when a real change in the measured construct (i.e., cognition) is unlikely (62). The assumption is that while between-person differences in scores on a given measure are expected, the score for any individual will remain constant across successive administrations of the instrument. In our population of interest, persons with TBI, there is evidence for the test-retest reliability of the HVLT-R and the ANAM4. One study (83) reported the Pearson correlation coefficient (PCC) and the other (68) reported the intra-class correlation coefficient (ICC), the preferred statistic. The HVLT-R was administered twice to 75 adults with TBI of unknown injury severity (71% men, 46.5 ± 10.5 years of age, at 11.8 ± 9.6 years post injury), the two sessions occurring 6–8 weeks apart from one another (83). Correlation coefficients for two of the eight HVLT-R scores (total recall and delayed recall) reflected high test-retest reliability (*r* = 0.82) and the remaining six scores (T-score, delayed recall T-score, retention, retention T-score, recognition discrimination index and recognition discrimination index T-score) reflected moderate (*r* = 0.64) test-retest reliability. The ANAM4 was administered twice to 1,324 members of the Marine Corps unit (all men, 22.5 ± 3.4 years of age) with a known high rate of concussion from combat and blast exposure. The average interval between the two test sessions was 357 ± 88 days (range 99–637 days) (68). After injury classification, 238 members were designated to the concussed group and 264 to the non-concussed group. While there were no significant differences between the mean scores of the two groups at the first session, differences emerged at second session, with the concussed group having lower mean scores than the non-concussed group on the cognitive tasks assessing attention, memory, spatial processing, reaction time, and cognitive fatigue [i.e., code substitution delayed (CDD), matching to sample (M2S), procedural reaction time (PRT), and simple reaction time (repeat) (SRT, SRT2) subscales]. The test of simple effects revealed that the mean score for the concussed group decreased significantly from T1 to T2 on the SRT, SRT2, PRT, Code Substitution Learning, M2S, Mathematical Processing (MTH), and CDD subscales. The ICC between the scores from the first and second sessions was reported only for the non-concussed group: of the seven domains, the CDD and MTH domains met the cut-off for the mean score correlation between the two time points for the entire group (i.e., >0.70) but not in the comparison of scores of individual patients at the two time points (i.e., >0.90). Practice effects were reported for the ANAM, where it was noted that individuals with TBI displayed inconsistent performance in 30 administrations over four days, while controls showed consistent improvement (85) (Table 3).

**Table 3:** Summary of domains assessed in instruments measuring cognitive functioning, and interpretation of scores.

**Measures**	**Neurocognitive Domains**						
	**Learning and memory**	**Language**	**Perceptual-motor**	**Complex attention**	**Executive function**	Information processing speed, reaction time^*^	**Social cognition**
ANAM	CDS, CDD—higher score is better		SPA, MTS—higher score is better	CDS, CDD, PRO– higher score is better	MTH, PRO, CDS, CDD, CPT—higher score is better	ST2, ST4, PRO, SRT, SRT2—higher score is better GNG—lower score is better	
ImPACT	WM, WM-D, VERM, DM, DM-D, VISM—higher score is better		DM, DM-D, VISM—higher score is better	TL– higher score is better CL, XO ICC; XO—lower score is better	SM, TL, VERM, VISM—higher score is better CL, XO ICC; XO, CL, SM, RT—lower score is better	SM, TL, VERM, VISM—higher score is better CL, XO ICC; XO, CL, SM, RT—lower score is better	
HVLT	Higher score is better				Higher score is better		
TMT			TMT A/B—lower score is better	TMT A/B—lower score is better	TMT A/B—lower score is better	TMT A/B—lower score is better	
PASAT				Higher score is better	Higher score is better	Higher score is better	
WAIS Total IQ—higher score is better	WMS—higher score is better	VCI, VIQ—higher score is better	PRI, PIQ—higher score is better	SS, CODE/DSST, CAN, VIQ, PIQ—higher score is better	VCI, PRI, WMS, VIQ, PIQ—higher score is better	PSS—higher score is better	Crystallized intelligence (use of learned knowledge) –higher score is better
FIM-COG Total score—higher score is better	Mem	Expr, socint—higher score is better			Comp, expr, socint, probsolv, mem—higher score is better		Higher score is better
CVLT	Higher score is better				Higher score is better		
COWAT	Higher score is better	Higher score is better		Higher score is better	Higher score is better		
RAVLT	Recall—higher score is better Forgetting—lower score is better Learning—higher score is better				Information retention– higher score is better Information retrieval– higher score is better		
ROCF	Higher score is better		Higher score is better		Higher score is better		
MMSE Total score—higher score is better	Recall—higher score is better Lang—higher score is better		Orient, visconst—higher Score is better		Regist, att and calc—higher score is better		
SCWT			Scoring varies		Scoring varies	Scoring varies	
SDMT			Higher score is better	Higher score is better		Higher score is better	
WMS Total MQ—higher score is better	LM, VR—higher score is better	PCI, ORI—higher score is better	ORI, VR—higher score is better		PCI, MC, MS, AL—higher score is better		

*Learning and memory: immediate memory, recent memory (free recall, cued recall, recognition memory), very-long-term memory (semantic, autobiographical), implicit learning*.

*Language: expressive language (naming, word finding, fluency, grammar, syntax), receptive language*.

*Perceptual-motor: visual perception, visuoconstructional, perceptual-motor, praxis, gnosis*.

*Complex attention: sustained attention, divided attention, selective attention, processing speed*.

*Executive function: planning, decision making, working memory, responding to feedback/error correction, overriding habits/inhibition, mental flexibility*.

*Social cognition: recognition of emotions, theory of mind*.

* *Information processing speed and reaction time: not part of DSM-5 classification; additional for our purposes*.

*AUTOMATED NEUROPSYCHOLOGICAL ASSESSMENT METRICS (ANAM): Sternberg memory search (ST2 and ST4), Mathematical processing (MTH), Spatial processing (SPA), Procedural reaction time (PRO), Simple reaction time (SRT), Simple reaction time repeat (SRT2), Code substitution (CDS), Code substitution-delayed (CDD), Continuous performance (CPT), Matching to sample (MTS), Go-no-go (GNG); IMMEDIATE POST-CONCUSSION ASSESSMENT AND COGNITIVE TESTING (ImPACT): Word memory (WM), Word memory-delayed (WM-D), Symbol match (SM), Three letters (TL), X's and O's (XO), Design memory (DM), Design memory-delayed (DM-D), XO (total correct answers on memory or distractor tasks), TL (avg counted correctly), Color match (CL); HOPKINS VERBAL LEARNING TEST (HVLT); TRAIL-MAKING TEST (TMT): Trails A (sequential connecting of numbers), Trails B (sequential connecting of numbers and letters); PACED AUDITORY SERIAL ADDITION TEST (PASAT); WECHSLER ADULT INTELLIGENCE SCALE (WAIS): Similarities (SIM), Vocabulary (VOCAB), Information (INFO), Comprehension (COMP), Block design (BD), Matrix reasoning (MR), Visual puzzles (VP), Picture completion (PC), Picture arrangement (PA), Figure weights (FW), Digit span (DSPAN), Arithmetic (ARIT), Letter-number sequencing (LNS), Symbol search (SS), Coding/digit symbol (CODE/DSST), Cancellation (CAN), Verbal intelligence (VIQ), Performance intelligence (PIQ), Full scale intelligence (FSIQ); FUNCTIONAL INDEPENDENCE MEASURE COGNITIVE DOMAIN(FIM-Cog): Comprehension (auditory), Expression (verbal), Social interaction, Problem solving, Memory; CALIFORNIA VERBAL LEARNING TEST (CVLT): Word list A (target words verbally delivered, 5 trials to learn), Word list B (distractor words); CONTROLLED ORAL WORDS ASSOCIATION TEST (COWAT); RAY AUDITORY VERBAL LEARNING TEST (RAVLT); REY-OSTERRIETH COMPLEX FIGURE TEST (ROCF); MINI-MENTAL STATE EXAMINATION (MMSE); STROOP COLOR-WORD TEST (SCWT); SYMBOL DIGIT MODALITIES TEST (SDMT); WECHSLER MEMORY SCALE (WMS)*.

### Responsiveness

Responsiveness, defined as the ability of an instrument to detect change over time in the construct being measured (62), was reported for the ANAM4 in a sample of concussed males (68) and for the WAIS in a sample of severe TBI (84). The former study of young men from the Marine Corps unit (68) tested the rate of performance decline on ANAM4 subscales from the first test session to the second, applying the reliable change (RC) methodology (86). Researchers reported that 48% of the concussed group demonstrated a decrease in performance on two or more subscales, compared to 28% of the non-concussed group.

When the WAIS was administered to 40 adults who sustained TBI and experienced posttraumatic amnesia (PTA) lasting at least 4 days (95% men, 28.3 ± 13.36 years of age) in the latter stages of their PTA and to a matched group of 40 non-injured persons, TBI group scores on the verbal subscales indicated less initial impairment and were restored to levels exhibited by the comparison group at a faster rate than were the scores on non-verbal subscales (84). The mean verbal intelligence quotient (IQ) of the TBI group approached that of the comparison group within the first year after injury, while performance IQ continued to improve over the course of 3 years (84, 87).

### Classification of Instruments of Cognitive Functioning by Cognitive Domain(s) Assessed

The WAIS assesses all seven cognitive domains, qualifying as a “global” measure of cognitive functioning. The remaining instruments were “multi-domain” measures. The most represented domain was learning and memory, assessed in 13 instruments, and the least represented was social cognition, included in two instruments (Table 3; Supplementary File 3).

### Information Relevant to Clinical and Research Applications

Instruments' manuals, assessment forms, and scoring instructions are available from their publishers; some are available online for free (Table 2). Information about the instruments (e.g., purpose, content, measurement properties, etc.) can be obtained online (Supplementary File 3).

All instruments require participants to complete one or more tasks, typically through written or spoken responses. The ANAM and ImPACT are computerized, the WAIS and WMS can be computer- or paper-based, and the FIM-Cog is administered via interview or participant observation. The number of items in each instrument varies: for instance, the COWAT presents three letters and relies on free word recall, while the PASAT contains a 61-item list of digits that the test-taker must sum up (i.e., adding each digit presented to the one that came just before it). The FIM-Cog, and larger batteries like the ANAM, ImPACT, WAIS and WMS, contain multiple tasks assessing different cognitive domains, ranging from five (e.g., FIM-Cog) up to 15 (e.g., WAIS), of which ten are core and five are supplementary subtests. Completion times range from 3 min (e.g., COWAT) to more than 90 min (e.g., ANAM, WAIS). Scoring procedures and score interpretation varies across instruments. Scoring sheets/software and instructions are available with purchase of the instruments.

### Evidence-Based Assessment of Cognitive Functioning Measures

All 15 instruments were utilized in at least two peer-reviewed studies by two different research teams. The WAIS and TMT were the most frequently used instruments: different versions of the WAIS were used 17 times by 14 different teams, and the TMT was used 14 times by 11 teams (Table 2). None of the instruments met the criteria for a “well-established,” “approaching well-established,” or “promising” rating in the TBI population (Table 3). Known-groups validity and responsiveness were reported for two versions of the ANAM in two concussion samples (53, 68). The hypotheses regarding known-groups validity were accepted for the HVLT-R delayed recall task but rejected for the total score in a mTBI sample (79), and test-retest reliability in a TBI sample of unknown severity met the correlation cut-off for the group, but not for individual patients for total recall and delayed recall only (83). Convergent and divergent construct validity hypothesis testing for other instruments were not supported for all subscales/tasks, and were not always in line with clinical expectations (69, 78).

Table 4 provides a summary of the measurement properties of measures of cognitive functioning in TBI samples.

**Table 4:** Summary of measurement properties of evaluative instruments of cognitive functioning in TBI samples.

**Instrument**	**Target population when developed**	**Neurocognitive domains covered**	Respondent burden^*^	**Construct validity**		**Ability to detect change**	
				**Convergent or divergent**	**Known-group**	**Test-retest reliability**	**Responsiveness**
ANAM	Healthy persons with environmental challenges	5/7	Up to 90 min	–	+	–	+
ImPACT	Athletes with concussion	5/7	25+ min	+	–	–	–
HVLT	General adult population	2/7	5–10 min testing + 25 min delay	–	+	+	–
TMT	Military personnel	4/7	5–10 min	–	+	–	–
PASAT	General population	3/7	15–20 min	+	–	–	–
WAIS	General adult population	7/7	60–90 min	+	+	–	+
FIM-COG	Rehabilitation in-patients	4/7	30–45 min	+	+	–	–
CVLT	General population	2/7	30 min testing + 30 min delay	+	–	–	–
COWAT	Persons with low education or limited writing	4/7	3+ min	–	+	–	–
RAVLT	General population	2/7	10–15 min	–	+	–	–
ROCF	Persons 6–89 years old	3/7	50–60 min	+	–	–	–
MMSE	Psychiatric and dementia patients	3/7	<5 min	+	–	–	–
SCWT	Psychiatric patients	3/7	5 min	+	–	–	–
SDMT	Persons 8 + years old with organic brain pathology	3/7	<5 min	–	+	–	–
WMS	General population	4/7	30–35 min	+	–	–	–

* *As reported for original version; +=information available in TBI sample(s) of mild severity; –=no information available in TBI samples of any severity*.

*COWAT, Controlled Oral Word Association Test; CVLT, California Verbal Learning Test; FIM, FIM instrument, cognitive subscale (formerly Functional Independence Measure); HVLT, Hopkins Verbal Learning Test; ImPACT, Immediate Post-concussion Assessment and Cognitive Test; MMSE, mini mental state examination; min, minutes; PASAT, Paced Auditory Serial Addition Test; RAVLT, Rey Auditory Verbal Learning Test; ROCF, Rey-Osterrieth Complex Figure Test; SDMT, Symbol Digit Modalities Test; TMT, Trail Making Test; WAIS (-R/III/IV), Wechsler Adult Intelligence Scale (-Revised/Third Edition/Fourth Edition); WMS (-R/III/IV), Wechsler Memory Scale (-Revised/Third Edition/Fourth Edition)*.

## Discussion

This review provides a comprehensive overview of existing instruments used to evaluate cognitive functioning in clinical and non-clinical settings in persons with TBI. An extensive search strategy led to identification of 15 instruments; each was reviewed and comprehensively described (Supplementary File 3), providing information on content and level of evidence existing regarding measurement properties as they concern the use of these tools for evaluative purposes, i.e., their ability to measure change in a construct over time. Our results highlight that most scientific evidence pertains to construct validity, with limited evidence on test-retest reliability and responsiveness in TBI samples. This poses a risk to TBI researchers and clinicians when it comes to interpreting the results produced in longitudinal studies, with no certainty of the instruments' ability to measure change in cognitive functioning in persons with TBI longitudinally. The results are informative nevertheless, having implications for the understanding of and future research related to the measurement properties of evaluative instruments, and their subsequent utilization for studying the natural history and clinical course of cognitive functioning and treatment effects in clinical trials in persons with TBI.

### Construct Validity

Construct validity refers to the development of a mini theory to describe how well an instrument measuring a construct of interest would agree with another instrument measuring a related construct (11, 62). In the measurement of cognitive functioning in persons with TBI, there does not exist a gold standard or criterion measure (11), and therefore understanding the domains each instrument is trying to measure (content validity) and whether the different instruments relate to one another in the way one would expect, is an important property to consider. It is important to highlight the basic principle of construct validation, which is that hypotheses about the relationship of scores of any instrument with scores on other instruments measuring a similar or different construct (convergent and divergent construct validity, respectively) should be formulated in advance; the specific expectations with regards to certain relationships can be based either on an underlying conceptual model or on the data in the literature (11). This review has found that only a few researchers tested hypotheses related to the relationship between the measures of cognitive functioning studies and measures of other constructs, stating ahead of analysis the expected direction and magnitude of associations, based on what was known about the constructs under study. In future studies, to assess similarity or dissimilarity between instruments' scores, when formulating hypotheses, one should first have a solid grasp of the contents of comparable instruments, which we provide in this review, as domains within any given instrument are expected to be correlated strongly with domains of conceptually similar instruments. There should also be a clear description of what is known about the TBI population under study, including but not limited to the circumstances surrounding injury, injury severity and mechanism, brain maturity and brain health at the time of injury, comorbid mental and physical disorders, coping ability, and psychotropic medication use, as each of these have the ability to influence cognitive functioning at assessment (88–92).

Known-groups validity (of construct validity) refers to an instrument's ability to discriminate between groups of individuals known to have a particular trait and those who do not have that trait (62). This property is most relevant for discriminative (i.e., diagnostic) instruments (11, 12), however is significant for evaluative instruments, where it is imperative that an instrument is responsive to all clinically important differences between constructs under investigation or different courses or outcomes of the construct (13). This includes identification and deletion of unresponsive items within a construct from the instrument over time. One way to identify such items within the cognitive functioning constructs assessed by instruments in the TBI population is to administer the instrument to a group of people with TBI of varying severities and associated cognitive impairments and to healthy people without impairments, and compare the scores yielded at baseline testing (known groups validity) and at follow-up after an intervention with known efficacy in improving cognition (e.g., cognitive training). Presence and absence of differences between the two groups in items will indicate items that are responsive and those that are not, respectively (longitudinal known-group construct validity). Only one study assessing measurement properties provided parameters of longitudinal known-group validity for the ANAM4 in young men with concussion (68) and those without.

### Test-Retest Reliability

Not every change on a measurement instrument can be considered a real or true change in the construct the instrument is believed to measure (13). Observed changes in scores over time may be due to measurement error, natural variability in a person's ability to concentrate throughout the day, i.e., peak in performance, mood at the time of investigation, which may determine positive or negative responses in case of doubt, evaluators' variability in applying criteria more or less strictly, or the natural course of the construct under study (i.e., recovery or deterioration) (11–13). Therefore, interpretation of change in score over time requires assessment of measurement error by test-retest of a stable population of interest (13). But what is a stable TBI population? By choosing a timeframe with 6–8 weeks between the two test sessions of the HVLT-R, researchers reported moderate to high test-retest reliability on all eight parameters tested (83). Unfortunately, the researchers did not ask their participants how their cognitive activity changed over the 6–8 weeks, and therefore, the stability of the group of TBI patients that took part in this study is unknown and thus the interpretability of the score and corresponding attribution of functional status is uncertain. When the researchers assessed test-retest reliability of the ANAM4 in young men in the chronic stage post-concussion and in their non-injured counterparts, the groups' scores were comparable at baseline, but differences in some but not all subscales (CDD, M2S, PRT, SRT, and SRT2) emerged at 357 ± 88 days after baseline assessment, with the concussed group exhibiting lower scores than the non-concussed group; the reason for this observation is not clear (68). Consequently, issues related to the meaning and interpretation of results of longitudinal studies utilizing instruments with unknown test-retest reliability in the population of interest remain pressing.

### Responsiveness

Responsiveness, defined as an instrument's ability to accurately detect change when it has occurred, is key for instruments applied for the purpose of evaluation (13). Responsiveness is not an attribute of the instrument itself, but reflects the application of the instrument in a given context (e.g., for quantifying the benefit of an intervention in a clinical trial), or to a certain type of change (i.e., natural history, recovery, etc.). Responsiveness is the ability of an instrument to detect change when it has taken place, and can be expressed either as absolute difference within a person or group, or the effect size, referred to as the standardized response mean (91). Responsiveness is the least studied attribute of measures of cognitive functioning. The results of one study that investigated responsiveness of the WAIS in a sample with severe head injury supported the hypothesis of differential speed of recovery of different domains of cognition in TBI (i.e., verbal IQ of the head injured group approached that of the comparison group within about one year of injury, while recovery of performance IQ continued to improve over about 3 years) (84). The results of another study assessing the ANAM4 in a concussed sample (68) reported that 48% of the concussed group demonstrated decreases in performance on two or more subscales compared with 28% of the non-concussed group, driven by the CDS, CDD, PRO, SRT, and ST2 subscales (assessing abilities related to information processing speed, reaction time, attention, memory, and learning). Neither study however, formulated specific hypotheses with respect to expected mean differences in scores in the studied groups a priori. Without the a priori hypotheses the risk of bias is high, because retrospectively, it is tempting to think of explanations for the observed results instead of concluding that an instrument might not be responsive. Also worth noting is that when measurement properties were assessed, participant samples consisted of mostly or strictly men. Without gender-related specifications on the applications of the ANAM and the HVLT, it impossible to draw any firm conclusions on the instruments' test-retest reliability and responsiveness, and therefore their ability to detect change over time.

### Feasibility, and Clinical and Research Utility

Feasibility concerns practicality of administering an instrument to a person in the setting it which it needs to be administered (11). In order to accurately measure what it intends to measure, and ensure valid responses, self-administered instruments must be completely self-explanatory, and those administered by research or clinical personnel require personnel to be trained to collect the required information. None of the studies included in this review reported on the training of respondents and administrators, or lack thereof. This is important for ensuring the procedure is standardized and to confirm the ability of persons with TBI to complete the procedure and respond with insight. Instruments that require more than 30 min to complete can be tiring for persons with TBI, who commonly experience decreased stamina, especially during tasks involving novelty. Multi-domain instruments demand continuous, goal-directed activity, which would be affected by diminished motivation, impairments, psychological state, and age. In such cases, researcher or clinicians may administer combinations of relevant sub-tests over some time, which can present a challenge for calculation of an accurate score and interpretation of scores. A composite score is the most practical approach for researchers looking to quantify a population's global change over time: if sample sizes are sufficiently large, the between-person variation in certain subscales or domains will be balanced in the calculation of a mean global score for the group. In the clinical setting, however, where clinicians are working one-on-one with individuals to study the natural history of or intervention-induced changes in a construct over time, focus on the individual subscales is key. The implication is that the scores of each of the subscales of a global or multi-domain measure have to be validated separately in a population of interest. This is particularly relevant for certain domains of cognition such as crystalized intellectual abilities, which have been hypothesized to be resistant to the effects of TBI. Finally, study of the evaluative properties of measures of cognitive functioning that are reflective of everyday cognitive skills is needed.

## Study Limitations

There are a number of limitations to this review. All of the studies included were published in English, and therefore instruments used in non-English-speaking populations and studies have not been captured. The review team did not contact authors of reviewed papers for additional methodological details that were not available in their publications. Further, the team appraised methodological quality utilizing Holmbeck et al.'s criteria (20), developed for measures of psychosocial adjustment and psychopathology, and therefore not specific for measures of cognitive functioning. Nevertheless, justifications for quality ratings given to each of the reviewed instruments were reported to clarify the resultant assessment grades. Several instruments [i.e., standardized assessment of concussion (SAS), short orientation-memory-concentration test (SOMT), and the neuropsychological assessment battery (NAB)] assessing orientation (i.e., time and place), were not reviewed in this work, as they were utilized just once within the articles identified in the primary search, and therefore did not meet the frequency of use criteria set for this review.

Another potential issue is that while the instruments themselves are standardized, there are a number of scores that could be derived from each one. For instance, CVLT scores can be based on the number of words recalled from multiple lists, recall after short/long delay, number of errors in recall, etc. The use of these scores was not consistent from study to study. This lack of consistency not only makes it challenging, if impossible, to compare scores across studies, but may impact evaluation of certain measurement properties, such as concurrent validity, if only certain scores are associated with instruments meant to measure similar/different constructs (Table 3; Supplementary File 3). There are limitations related to the identification of data on construct validity. For the purpose of this review, data on measurement properties was gathered from studies of longitudinal design only. Despite attempts to include all articles relevant to construct validity in the TBI population, it is possible some cross-sectional studies evaluating construct validity were missed.

Finally, while the potential application of the described instruments measuring cognitive functioning in TBI can be for diagnostic/descriptive and prognostic/predictive purposes, the focus of our work was to examine the evaluative properties of such instruments (i.e., their ability to measure the magnitude of change longitudinally), when no external criterion is available for validating the construct. Thus, the assessment of properties of instruments included in this review, as descriptive or predictive measures of cognitive functioning, requires further study.

## Conclusions

In research utilizing evaluative, or other, psychometrics, the suitability of the instruments, or lack thereof, in terms of their psychometric properties, is rarely discussed, or acknowledged as a limitation in the case of measures whose scores have not been validated. This review highlights the problematic use of certain measures that lack the properties necessary for their use as evaluative measures. The evidence on measurement properties of instruments used to assess cognitive functioning in TBI samples longitudinally is limited, and thus, the way forward appears to be consensus on a set of measures with the greatest potential for evaluative purposes in TBI, and assessment of these select measures to build the evidence on measurement properties and establish or refute rationale for their application in TBI research. Refinement and testing of this group of instruments in TBI samples of varying severities in terms of longitudinal construct validity, test-retest reliability, and responsiveness, not only to reliably study the course of cognitive functioning after brain injury, but also for quantifying treatment benefits in clinical trials, is timely. Assessment of psychometric properties should not be an afterthought, but rather should preface the application of a measure in any new population or context, serving as the deciding factor on whether to proceed with its use. It is important that future research on psychometric properties of evaluative psychometrics, take into account the heterogeneity in cognitive functioning of persons with TBI, and report stratified results for subgroups of people based on injury severity, mechanism, and baseline cognitive abilities, to mitigate some of the heterogeneity. Further, to present these results in the context of change in everyday functional capabilities as time since injury progresses or in response to intervention, which will provide valuable insights. Furthermore, emphasis on within-individual variability in the TBI population, where each person serves as their own control, is likely to be the best technique to analyse change, and to answer the question—to which extent is the inherent regenerative capacity affected by injury-related variables, as opposed to internally (age, sex, genetic profile, etc.) or environmentally driven variables, as well as brain-behavior relations. The trade-off with the latter, however, is limited standardization of individual outcome measures, and lack of current theories of psychometric properties that speak to single case experimental design studies only (relevant to personalized medicine theory) and limited external validity (i.e., generalizability).

## Ethics Statement

This article does not contain any studies with human or animal subjects performed by any of the authors.

## Author Contributions

TM and AC contributed to the conception of the study. TM developed the idea, registered the review on PROSPERO, designed and published the protocol, and developed the study screening criteria and quality assessment criteria. TM, SM, NP, FJ and AD executed the study in accordance with the protocol protocol. AD and NP screened all abstracts. AD extracted the data. SM doubled checked all extracted data. AD, SM, and NP performed study quality assessment and abstracted the data. TM guided the process and checked all data. AD and SM performed data analyses. AD wrote the first draft of the review, which was then edited by SM and TM. TM and AC provided mentorship to AD, SM, NP, and FJ throughout the course of the study.

### Conflict of Interest Statement

The authors declare that the research was conducted in the absence of any commercial or financial relationships that could be construed as a potential conflict of interest. The handling editor declares a shared association, though no other collaboration, with one of the authors AC in the Canadian concussion consortium.

## Figures and Tables

**Table:** Glossary of terms adapted from Feinstein (11), Kirshner and Guyatt (12), and the COSMIN [Mokkink et al. (62)] definitions.

Discriminative instruments	Measures used to distinguish between individuals or group on an underlying dimension when no external criterion or gold standard is available for validating these measures. Key psychometric properties: construct validity (differentiate high/low levels, acts as expected) and reliability (internal consistency, inter-rater reliability)
Evaluative instruments	Measures used to assess the magnitude of longitudinal change in an individual or group on the dimension of interest. Key psychometric properties: construct validity (measures target construct), test-retest reliability, and responsiveness to change
Predictive instruments	Measures used to classify individuals into a set of predefined measurement categories when a gold standard is available, either concurrently or prospectively, to determine whether individuals have been classified correctly. Key psychometric properties: construct validity (measures target construct, predicts future events), reliability (internal consistency, inter-rater reliability), statistical association with gold standard (criterion measure)
Content validity	The degree to which an instrument adequately samples from the domain of interest
Construct validity	The degree to which the measure scores reflect the hypotheses; includes (1) convergent, (2) divergent, and (3) known-group validity
Convergent validity	The degree of relatedness between two constructs hypothesized to be related
Divergent validity	The degree of relatedness Between two constructs hypothesized to be different
Known-group validity	Ability of the measure to discriminate between group of individuals known to have a particular trait and those who do not have that trait (same as discriminative validity)
Reliability	The extent to which the measure is reliable, that is, free of errors in score not due to true state of construct measured in the patient; consists of (1) internal consistency, (2) test-retest, (3) inter-rater, and (4) intra-rater
Inter-rater reliability	Degree of agreement between the score given by one rater and that by another at one time with respect to the same respondent; addresses the interpretability of the measure; falls under the broader test-retest reliability category
Intra-rater reliability	Degree of agreement between scores given by the same respondent or rater at one time and those given at another time; falls under the broader test-retest reliability category
Feasibility	Practicality of administering the measure; completion time, and scoring formula
Responsiveness	Ability of an instrument to detect change over time in the construct to be measured

## Abbreviations

ANAM: Automated Neuropsychological Assessment Metrics
CNS: Central Nervous System
COWAT: Controlled Oral Word Association Test
CVLT: California Verbal Learning Test
DSM-5: Diagnostic and Statistical Manual of Mental Disorders-Fifth Edition
FIM-Cog: Functional Independence Measure-Cognitive Subscale
GCS: Glasgow Coma Scale
HVLT: Hopkins Verbal Learning Test
ImPACT: Immediate Post-concussion Assessment and Cognitive Test
MMSE: Mini-mental State Examination
PASAT: Paced Auditory Serial Addition Test
PROSPERO: International Prospective Register of Systematic Reviews
QUIPS: Quality in Prognosis Studies
RAVLT: Rey Auditory Verbal Learning Test
ROCF: Rey-Osterrieth Complex Figure Test
SDMT: Symbol Digit Modalities Test
SIGN: Scottish Intercollegiate Guidelines Network
TBI: Traumatic Brain Injury
TMT: Trail Making Test
WAIS: Wechsler Adult Intelligence Scale
WMS: Wechsler Memory Scale.
