# Supplementary material for: Measuring Change Over Time: A Systematic Review of Evaluative Measures of Cognitive Functioning in Traumatic Brain Injury
Source: Front Neurol. 2019 May 8;10:353. doi: 10.3389/fneur.2019.00353 (PMC6517520; doi:10.3389/fneur.2019.00353)
Supplement: Supplementary file 1 [file Data_Sheet_1.docx]

**Supplementary File 1**. Database search strategy

Database: Ovid MEDLINE(R) ALL <1946 to December 24, 2017>

Search Strategy:

--------------------------------------------------------------------------------

1 AUTOMATED NEUROPSYCHOLOGICAL ASSESSMENT METRIC?.af. (125)

2 ANAM.ti,ab,kf. (170)

3 "IMMEDIATE POST-CONCUSSION ASSESSMENT AND COGNITIVE TEST???".af. (167)

4 [impact.ti,kw.] (0)

5 HOPKINS VERBAL LEARNING TEST.af. (313)

6 HVLT.ti,ab,kf. (160)

7 Trail Making Test/ (731)

8 TRAIL-MAKING TEST???.af. (3114)

9 TMT.ti,ab,kf. (2547)

10 PACED AUDITORY SERIAL ADDITION TEST???.af. (394)

11 PASAT.ti,ab,kf. (456)

12 WECHSLER ADULT INTELLIGENCE SCAL*.af. (1840)

13 WAIS.ti,ab,kf. (2704)

14 (FUNCTIONAL INDEPENDENCE MEASURE adj10 COGNIT*).af. (184)

15 (FIM adj10 COG*).ti,ab,kf. (416)

16 CALIFORNIA VERBAL LEARNING TEST???.af. (892)

17 CVLT.ti,ab,kf. (573)

18 CONTROLLED ORAL WORD? ASSOCIATION TEST???.af. (282)

19 COWAT.ti,ab,kf. (103)

20 REY AUDITORY VERBAL LEARNING TEST???.af. (691)

21 RAVLT.ti,ab,kf. (342)

22 REY OSTERRIETH COMPLEX FIGURE TEST???.af. (243)

23 ROCF?.ti,ab,kf. (198)

24 MINI-MENTAL STATE EXAM*.af. (12283)

25 MMSE.ti,ab,kf. (9394)

26 (STROOP adj3 TEST???).af. (3455)

27 Stroop Test/ (1373)

28 SCWT.ti,ab,kf. (93)

29 SYMBOL DIGIT MODALITIES TEST???.af. (555)

30 SDMT.ti,ab,kf. (366)

31 Wechsler Memory Scale/ (8)

32 wechsler memory scal*.af. (1304)

33 WMS.ti,ab,kf. (1195)

34 or/1-33 (30405)

35 exp brain injuries/ (62041)

36 Craniocerebral Trauma/ (21290)

37 exp Brain Injuries, Traumatic/ (9549)

38 (TBI or mTBI or wrTBI).tw,kf. (22048)

39 (concuss* or postconcuss*).tw,kf. (8081)

40 ((brain* or cerebr* or intracerebr* or crani* or intracran* or head* or subdural* or epidural* or extradural*) adj (haematoma* or hematoma* or hemorrhag* or haemorrhag* or bleed*)).tw,kf. (46530)

41 ((head* or cerebr* or crani* or skull* or intracran*) adj2 (injur* or trauma* or damag* or wound* or swell* or oedema* or edema* or fracture* or contusion* or pressur*)).tw,kf. (80329)

42 or/35-41 (178066)

43 34 and 42 (1385)

44 43 not ((exp infant/ or exp children/ or exp adolescent/) not (exp adult/ or exp aged/)) (1284)

45 Validation Studies.pt. (90254)

46 exp Psychometrics/ (67725)

47 (validation or validity).tw,kf. (298152)

48 exp "Reproducibility of Results"/ (360234)

49 exp Discriminant Analysis/ (9465)

50 (reliab* or unreliab* or valid* or coefficient or homogeneity or homogeneous or "internal consistency").tw,kf. (1211585)

51 (agreement or precision or imprecision or "precise values" or test-retest).tw,kf. (368896)

52 (test and retest).tw,kf. (23488)

53 (reliab* and (test or retest)).tw,kf. (78735)

54 exp "Sensitivity and Specificity"/ (528853)

55 exp Longitudinal Studies/ (116680)

56 (longitudinal* or responsiv*).tw,kf. (434643)

57 or/45-56 (2437359)

58 44 and 57 (517)

59 remove duplicates from 58 (517)

| **Supplementary File 2**. Quality assessment of studies. | | | | | | | | | | | | | | | | | | | | | | | | | | |
| --- | --- | --- | --- | --- | --- | --- | --- | --- | --- | --- | --- | --- | --- | --- | --- | --- | --- | --- | --- | --- | --- | --- | --- | --- | --- | --- |
| Study | Study Participation | | | | Study Design | | | | Study Attrition | | | | | | Prognostic Factors | | | | | Confounding Measurement | | Outcome Measure | | | | Overall |
|  | Key characteristics | Inclusion/exclusion | Key characteristics BS/FU | # eligible participants | Pro/retro | Sampling frame | Recruitment | Place of recruitment | % | Description Adequacy | Attempt to collect  Reasons for lost |  | Characteristics of lost | No difference b/w participants and lost | Definition provided | Reliable method | Same setting | Missing data reported | Imputation | Minimum Confounders | Appropriate Analysis | Outcome definition | Duration FU | Valid/reliable measure | Same method/setting |  |
| Bleiberg et al. (2004) | no | yes | yes | no | pro | no | no | no | no | N/A | N/A | N/A | N/A | N/A | N/A | N/A | N/A | N/A | N/A | N/A | N/A | no | no | unsure | no | (++) |
| Chen et al. (2013) | no | partly | yes | no | pro | no | no | no | no | N/A | N/A | N/A | N/A | N/A | N/A | N/A | N/A | N/A | N/A | N/A | N/A | no | no | unsure | yes | (+) |
| Christensen et al. | no | no | yes | yes | pro | unsure | no | no | no | N/A | N/A | N/A | N/A | N/A | NA | NA | NA | NA | NA | N/A | N/A | no | no | unsure | no | (+) |
| Covassin et al. (2012) | no | no | no | yes | pro | no | no | no | no | N/A | N/A | N/A | N/A | N/A | N/A | N/A | N/A | N/A | N/A | N/A | N/A | no | no | unsure | no | (++) |
| Covassin et al. (2007) | yes | partly | yes | yes | pro | unsure | no | no | no | N/A | N/A | N/A | N/A | N/A | NA | NA | NA | NA | NA | N/A | N/A | no | no | unsure | no | (+) |
| Covassin et al. (2008) | no | yes | yes | yes | pro | no | no | no | no | N/A | N/A | N/A | N/A | N/A | N/A | N/A | N/A | N/A | N/A | N/A | N/A | no | no | unsure | no | (+) |
| Dikmen et al. (2017) | no | no | yes | yes | pro | no | no | no | no | yes | yes | yes | yes | yes | N/A | N/A | N/A | N/A | N/A | N/A | N/A | no | no | unsure | unsure | (+) |
| Failla et al. (2015) | no | no | yes | no | pro | unsure | no | no | no | no | partly | partly | yes | unsure | no | no | no | unsure | unsure | yes | yes | no | no | unsure | no | (+) |
| Farbota et al. (2012) | no | no | no | unsure | pro | unsure | no | no | no | no | no | no | no | unsure | N/A | N/A | N/A | N/A | N/A | N/A | N/A | no | no | unsure | no | (++) |
| Field et al. (2003) | no | no | yes | unsure | pro | no | no | no | no | N/A | N/A | N/A | N/A | N/A | N/A | N/A | N/A | N/A | N/A | N/A | N/A | no | no | unsure | no | (++) |
| Kersel et al. (2001) | no | no | partly | no | pro | no | no | no | no | N/A | N/A | N/A | N/A | N/A | N/A | N/A | N/A | N/A | N/A | N/A | N/A | no | no | unsure | no | (++) |
| Kontos et al. (2015) | no | no | no | yes | pro | no | no | unsure | no | N/A | N/A | N/A | N/A | N/A | N/A | N/A | N/A | N/A | N/A | N/A | N/A | no | no | unsure | unsure | (+) |
| Kwok et al. (2008) | no | no | yes | yes | pro | no | no | no | no | yes | yes | yes | partly | unsure | N/A | N/A | N/A | N/A | N/A | N/A | N/A | no | no | unsure | no | (+) |
| Lee et al. (2005) | no | no | yes | yes | pro | yes | no | no | no | N/A | N/A | N/A | N/A | N/A | N/A | N/A | N/A | N/A | N/A | N/A | N/A | no | no | unsure | no | (+) |
| Liberman et al (2002) | no | no | partly | no | pro | no | no | no | no | N/A | N/A | N/A | N/A | N/A | N/A | N/A | N/A | N/A | N/A | N/A | N/A | no | no | unsure | no | (++) |
| Losoi et al. (2016) | no | no | partly | no | pro | no | no | no | no | N/A | N/A | N/A | N/A | N/A | N/A | N/A | N/A | N/A | N/A | N/A | N/A | no | no | unsure | no | (++) |
| Macciocchi et al. (2004) | no | no | yes | no | retro | no | no | no | no | N/A | N/A | N/A | N/A | N/A | N/A | N/A | N/A | N/A | N/A | NA | NA | no | no | unsure | no | (+) |
| Maksymiuk et al. (2005) | no | no | yes | yes | retro | no | no | no | no | N/A | N/A | N/A | N/A | N/A | N/A | N/A | N/A | N/A | N/A | N/A | NA | no | no | unsure | no | (+) |
| Mandleberg (1976) | no | no | no | yes | pro | unsure | no | no | no | N/A | N/A | N/A | N/A | N/A | N/A | N/A | N/A | N/A | N/A | N/A | N/A | no | no | unsure | no | (+) |
| McCrea et al. (2005) | partly | no | yes | no | pro | no | no | no | no | N/A | N/A | N/A | N/A | N/A | N/A | N/A | N/A | N/A | N/A | N/A | NA | no | no | unsure | no | (+) |
| Meier et al. (2015) | no | partly | yes | yes | pro | no | no | no | no | N/A | N/A | N/A | N/A | N/A | N/A | N/A | N/A | N/A | N/A | N/A | NA | no | no | unsure | no | (+) |
| Ponsford et al. (2011) | no | no | yes | no | pro | no | no | no | no | no | no | no | yes | unsure | N/A | N/A | N/A | N/A | N/A | N/A | N/A | no | no | unsure | no | (+) |
| Powell et al. (1996) | no | no | no | unsure | pro | no | no | no | no | yes | yes | yes | no | no | N/A | N/A | N/A | N/A | N/A | N/A | N/A | partly | no | unsure | unsure | (+) |
| Prigatano et al. (1984) | no | yes | yes | yes | retro | no | no | no | no | N/A | N/A | N/A | N/A | N/A | N/A | N/A | N/A | N/a | N/A | N/A | N/A | no | no | unsure | unsure | (+) |
| Register-Mihalik et al. (2013) | no | yes | yes | unsure | pro | no | no | partly | yes | yes | yes | yes | yes | unsure | N/A | N/A | N/A | N/A | N/A | N/A | N/A | no | no | no | unsure | (+) |
| Robertson et al. (2015) | no | no | no | yes | pro | yes | no | no | no | yes | yes | yes | no | unsure | no | no | unsure | unsure | N/A | no | no | no | no | unsure | unsure | (+) |
| Sandhaug et al. (2015) | no | partly | yes | yes | pro | no | no | no | no | yes | yes | yes | yes | no | N/A | N/A | N/A | N/A | N/A | N/A | N/A | no | no | unsure | no | (+) |
| Schmitter et al. (2015) | no | no | no | yes | pro | yes | no | no | no | yes | yes | yes | no | no | N/A | N/A | N/A | N/A | N/A | N/A | N/A | no | no | unsure | unsure | (+) |
| Snow et al. (1998) | no | no | no | unsure | pro | unsure | unsure | no | no | no | no | no | yes | unsure | N/A | N/A | N/A | N/A | N/A | N/A | N/A | no | no | no | unsure | (+) |
| Sosnoff et al. (2008) | no | yes | yes | unsure | pro | no | no | no | no | N/A | N/A | N/A | N/A | N/A | N/A | N/A | N/A | N/A | N/A | N/A | N/A | no | no | unsure | unsure | (+) |
| Sours et al. (2015) | no | no | yes | unsure | pro | unsure | no | no | no | N/A | N/A | N/A | N/A | N/A | no | unsure | no | no | N/A | yes | yes | no | no | partly | no | (++) |
| Till et al. (2008) | no | no | yes | no | pro | yes | no | no | no | N/A | N/A | N/A | no | unsure | N/A | N/A | N/A | N/A | N/A | N/A | N/A | no | no | unsure | unsure | (+) |
| Tofil & Clinchot. (1996) | no | partly | no | yes | pro | unsure | no | no | no | N/A | N/A | N/A | N/A | N/A | N/A | N/A | N/A | N/A | N/A | N/A | N/A | no | no | unsure | no | (+) |
| Vanderploeg et al. (2014) | no | no | yes | yes | pro | unsure | no | no | no | N/A | N/A | N/A | N/A | N/A | N/A | N/A | N/A | N/A | N/A | N/A | N/A | no | no | unsure | no | (+) |
| Wang et al. (2013) | no | no | yes | yes | pro | yes | no | no | no | N/A | N/A | N/A | N/A | N/A | N/A | N/A | N/A | N/A | N/A | N/A | NA | no | no | unsure | unsure | (+) |
| Whyte et al. (2013) | no | partly | no | no | pro | no | no | no | no | N/A | N/A | N/A | N/A | N/A | N/A | N/A | N/A | N/A | N/A | N/A | N/A | no | no | unsure | yes | (+) |
| Wylie et al. (2015) | no | no | no | yes | pro | unsure | no | no | no | N/A | N/A | N/A | N/A | N/A | N/A | N/A | N/A | N/A | N/A | N/A | N/A | no | no | unsure | no | (++) |
| Zafonte et al. (2012) | no | no | no | no | pro | unsure | no | no | no | no | no | no | yes | yes | N/A | N/A | N/A | N/A | N/A | N/A | N/A | no | no | unsure | yes | (+) |

Yes – yes, sources of potential bias are presented

No – no potential bias

Unsure – not enough details were reported to make a decision

N/A - not applicable according to the study design or type of analyses used

**Supplementary File 3.** Characteristics of 15 selected instruments (ANAM, COWAT, CVLT, FIM-Cog, MMSE, HVLT, ImPACT, PASAT, RAVLT, ROCF, SDMT, Stroop Task, TMT, WAIS, and WMS)

**THE AUTOMATED NEUROPSYCHOLOGICAL ASSESSMENT METRICS (ANAM)**

| Descriptive | Method of Development | Developed as a clinical subset of OMPAT’s TWB cognitive processing library of tests; initially for use in healthy subjects w/ environmental challenges but adapted as brief assessment of cognition w/i clinical populations^1^ |
| --- | --- | --- |
|  | Purpose | To briefly assess patients w/ cognitive impairment^1^ over repeated testing^1-2^ |
|  | Content | Assesses sustained attention, working memory, visual search, spatial processing, computation, immediate and delayed reaction time, performance efficiency^3^, decision-making, executive function, concentration^4^  Modules: 2-CRT, CDS (Learning/Immediate/CDD), EM, G/NG, GR, LR (Symbolic), Manikin, MG, MTS, MTH, STN, PRO, PT, RMCPT, SRT, SPA (Sequential/Simultaneous), Standard CPT, Stroop, Switching, Tapping, TWP^5^ |
|  | Response – options | Responses to each module made using a computer and keyboard/mouse^6^ |
|  | Recall period | Not applicable |
|  | Endorsements | Available to individuals w/ appropriate qualification^4^ |
| Practical | To obtain | Available from Vista: http://www.vistalifesciences.com/order-anam^6^ |
|  | Method of administration | Patient must complete each module; individual descriptions of administration available for certain frequently used modules^2^ |
|  | Scoring and interpretation | APR software generates full report for subtests and compares to norms; ADEPT software extracts and organizes data^3-4^; common scores include % correct/AC; mean RT for accurate responses, and TP (accuracy to speed ratio)^7^ |
|  | Respondent burden | Few minutes (individual modules); up to 90+ minutes (entire battery)^7^ |
|  | Admin burden | Administrator must have knowledge of APA distribution and administration, guidelines^4^; must purchase software and obtain computer access^6^ |
|  | Translations | English^6^ |
| Critical Appraisal Value | Strengths, Cautions, Clinical and Research Applicability | Allows for generation of infinite alternative forms, randomization of stimuli, cognitive profile creation^8^, and subtle detection of RT changes w/ short administration time and automated analysis^7,8^  Not an ideal concussion screening tool beyond the first 72 hours post-injury^9^  Little established normative data;^8^ not extensively studied in TBI samples |

AC, accuracy; ADEPT, ANAM Data Extraction and Presentation Tool; ANAM, Automated Neuropsychological Assessment Metrics; APA, American Psychological Association; APR, ANAM Performance Report; CDD, Code Substitution Delayed Recall; CDS, Code Substitution; EM, Effort Measure; G/NG, Go/No-Go; GR, Grammatical Reasoning; LR, Logical Reasoning; MG, Matching Grids; MTH, Mathematical Processing; MTS, Matching to Sample; OMPAT, Office of Military Performance Assessment Technology; PRO, Procedural Reaction Time; RMCPT, Running Memory Continuous Performance Task; PT, Pursuit Tracking; RT, reaction/response time; SPA, Spatial Processing; SRT, Simple Reaction Time; STN, Sternberg Memory Search; TBI, traumatic brain injury; TP, throughput; TWB, Tester’s Workbench; TWP, Tower Puzzle; TBI-only population

**References**

1. Kane, R., Roebuckspencer, T., Short, P., Kabat, M., & Wilken, J. (2007). Identifying and monitoring cognitive deficits in clinical populations using Automated Neuropsychological Assessment Metrics (ANAM) tests. *Archives Of Clinical Neuropsychology*, *22*(Supplement 1), 115-126. http://dx.doi.org/10.1016/j.acn.2006.10.006
2. Kabat, M., Kane, R., Jefferson, A., & DiPino, R. (2001). Construct Validity of Selected Automated Neuropsychological Assessment Metrics (ANAM) Battery Measures. *The Clinical Neuropsychologist (Neuropsychology, Development And Cognition: Section D)*, *15*(4), 498-507. http://dx.doi.org/10.1076/clin.15.4.498.1882
3. Register-Mihalik, J., Guskiewicz, K., Mihalik, J., Schmidt, J., Kerr, Z., & McCrea, M. (2013). Reliable Change, Sensitivity, and Specificity of a Multidimensional Concussion Assessment Battery. *Journal Of Head Trauma Rehabilitation*, *28*(4), 274-283. http://dx.doi.org/10.1097/htr.0b013e3182585d37
4. *ANAM Software Suite*. *Vistalifesciences.com*. Retrieved 10 August 2017, from http://www.vistalifesciences.com/anam-software-suite
5. *ANAM Test Library*. *Vistalifesciences.com*. Retrieved 10 August 2017, from http://www.vistalifesciences.com/anam-test-library
6. *Order ANAM*. *Vistalifesciences.com*. Retrieved 10 August 2018, from http://www.vistalifesciences.com/order-anam
7. Ibarra, S. (2011). Automated Neuropsychological Assessment Metrics. In: *Encyclopedia of Clinical Neuropsychology*. Springer International Publishing, pp. 325-327.
8. Collie, A., Darby, D., & Maruff, P. (2001). Computerised cognitive assessment of athletes with sports related head injury. *British Journal Of Sports Medicine*, *35*(5), 297-302. http://dx.doi.org/10.1136/bjsm.35.5.297
9. Coldren, R., Russell, M., Parish, R., Dretsch, M., & Kelly, M. (2012). The ANAM Lacks Utility as a Diagnostic or Screening Tool for Concussion More Than 10 Days Following Injury. *Military Medicine*, *177*(2), 179-183. http://dx.doi.org/10.7205/milmed-d-11-00278
10. Bleiberg, J., Cernich, A.N., Cameron, K., Sun, W., Peck, K., Ecklund, P.J., Reeves, D., Uhorchak, J., Sparling, M.B., Warden, D.L. (2004). Duration of cognitive impairment after sports concussion. Neurosurgery, 54(5), 1073-78; discussion 1078-80.

**THE CONTROLLED ORAL WORD ASSOCIATION TEST (COWAT)**

| Descriptive | Method of Development | Developed to provide a short test that is also feasible for patients w/ lower education or limited use of writing hand^1^ |
| --- | --- | --- |
|  | Purpose | To evaluate verbal fluency, specifically phonemic fluency^1^ |
|  | Content | Assesses ability to retrieve information – selective attention, mental set shifting, response generation, and self-monitoring^1^ |
|  | Response – options | Patient must name words that begin with a given letter^1^ |
|  | Recall period | Not applicable |
|  | Endorsements | Available to individuals w/ appropriate qualification^2^ |
| Practical | To obtain | Available as part of the MAE battery from PAR: http://www4.parinc.com/Products/Product.aspx?ProductID=MAE^2^ |
|  | Method of administration | Patient is given 1 minute to name as many words as possible beginning with a certain letter; repeated for two other letters^1^; commonly used English letter sets are are CFL, PRW, and FAS^3^ |
|  | Scoring and interpretation | Overall: # words for all three letters w/o repetitions and proper nouns  Other possible measures include: error analysis (error patterns suggestive of executive dysfunction), Troyer clustering (extent of production of items within a given group/mean cluster size), Troyer switching (ability to switch to another group of words/# of switches)^1^, Abwender cluster switches (from one cluster to a related cluster), and Abwender hard switches (from a cluster to non-cluster words, or b/w non-cluster words^4^ |
|  | Respondent burden | 3 minutes to complete^3^ |
|  | Admin burden | Requires Level C qualification; must purchase test kit^2^ |
|  | Translations | English, Spanish^2^ |
| Critical Appraisal Value | Strengths, Cautions, Clinical and Research Applicability | Measurement properties not extensively studied in TBI populations  Significant predictors of score include education [F(2, 336) = 16.21; p < 0.0001]^3^and size of the corpus callosum (R^2^ = 0.14; p < 0.05) ^5^  Total score moderately correlated w/ education (r = 0.367; p < 0.01) but NS w/ age (r = -0.03; p > 0.05) ^6^ |

COWAT, Controlled Oral Word Association Test; MAE, Multilingual Aphasia Examination; PAR, Psychological Assessment Resources; TBI, traumatic brain injury

**References**

1. Patterson, J. (2011). Controlled Oral Word Association Test. In: *Encyclopedia of Clinical Neuropsychology*. Springer International Publishing, pp.703-706.
2. *MAE (Multilingual Aphasia Examination, 3rd Ed.)*. (2012). *www4.parinc.com*. Retrieved 1 August 2017, from http://www4.parinc.com/Products/Product.aspx?ProductID=MAE#Items
3. Ruff, R., Light, R., Parker, S., & Levin, H. (1996). Benton controlled oral word association test: Reliability and updated norms. *Archives Of Clinical Neuropsychology*, *11*(4), 329-338. http://dx.doi.org/10.1093/arclin/11.4.329
4. Abwender, D., Swan, J., Bowerman, J., & Connolly, S. (2001). Qualitative Analysis of Verbal Fluency Output: Review and Comparison of Several Scoring Methods. *Assessment*, *8*(3), 323-338. http://dx.doi.org/10.1177/107319110100800308
5. Rao, S., Leo, G., Haughton, V., Aubin-Faubert, P., & Bernardin, L. (1989). Correlation of magnetic resonance imaging with neuropsychological testing in multiple sclerosis. *Neurology*, *39*(2), 161-161. http://dx.doi.org/10.1212/wnl.39.2.161
6. Steinberg, B., Bieliauskas, L., Smith, G., Langellotti, C., & Ivnik, R. (2005). Mayo's Older Americans Normative Studies: Age- and IQ-Adjusted Norms for the Boston Naming Test, the MAE Token Test, and the Judgment of Line Orientation Test. *The Clinical Neuropsychologist*, *19*(3-4), 280-328. http://dx.doi.org/10.1080/13854040590945229

**THE CALIFORNIA VERBAL LEARNING TEST (CVLT)**

| Descriptive | Method of Development | Developed by Delis et al. in 1987 to assess learning and memory in terms of what is remembered, how items are remembered and what errors are made^1^ |
| --- | --- | --- |
|  | Purpose | To evaluate learning with respect to a verbally-presented list of words^1^ |
|  | Content | Assesses verbal learning and memory^2^  CVLT: contains 3 lists of shopping items from various semantic categories^2-3^; + List A (tools, fruits, clothing, and spices and herbs); List B (interference list consisting of fruits, spices and herbs – shared w/ List A – fish, and kitchen utensils – distinct); 44-item recognition list containing target words from List A and distractor words^3^  CVLT-II: 4 categories are semantically unrelated; optional 16-item forced choice discrimination task to measure effort; new alternate test form and new short form (9-item lists) for patients with severe cognitive dysfunction^4^ |
|  | Response – options | Recall: FR (patient must recall as many words as possible in any order); CR (examiner prompts patient w/ category name); can be SDFR/SDCR or 20-minute LDFR/LDCR  Recognition: patient must classify 44 words as targets or distractors^3^ |
|  | Recall period | Not applicable |
|  | Endorsements | Available to individuals w/ appropriate qualification^5^ |
| Practical | To obtain | CVLT-II available from Pearson Education: http://www.pearsonclinical.com/psychology/products/100000166/california-verbal-learning-test--second-edition-cvlt-ii.html^5^ |
|  | Method of administration | CVLT: examiner reads each list out loud with 1s intervals b/w words; 5 List A trials and 1 List B trial w/ immediate FR after each trial; List A SDFR/SDCR assessed immediately after the 6 trials; List A LDFR/LDCR assessed 20 minutes later; recognition list read and assessed at the end^2-3^  CVLT-II: forced choice discrimination task follows recognition task^4^ |
|  | Scoring and interpretation | Can be scored manually but accompanying computer software is encouraged^3^  CVLT: various scores possible, including # words correctly recalled per List A/B trial, # correctly recalled in SDFR/SDCR/LDFR/LDCR, and # RHs/FPs^2^  CVLT-II: primary measures are total trials 1-5, SDFR, SDCR, LDFR, LDCR, and total recognition discrim., w/ additional process measures^6^ |
|  | Respondent burden | CVLT-II: 30 minutes of testing + 30 minute delay period (standard from); 15 minutes of testing + 15 minute delay period (short from)^5^ |
|  | Admin burden | Requires Level C qualification; must purchase test kit and software^3^ |
|  | Translations | English, Italian, German^3^, Korean^7^ |
| Critical Appraisal Value | Strengths, Cautions, Clinical and Research Applicability | Demonstrated predictive validity in TBI populations  Moderate to high convergent validity and test-retest reliability but not widely studied in TBI populations |

CR, cued recall; CVLT/CVLT-II, California Verbal Learning Test/California Verbal Learning Test-Second Edition; FP, false positives; LDCR, long delay cued recall; LDFR, long delay free recall; LDR, long delay recall; NS, non-significant/non-significantly; RAVLT, Rey Auditory Verbal Learning Test; RH, recognition hits; SDCR, short delay cued recall; SDFR, short delay free recall; TBI; traumatic brain injury

**References**

1. Yi, A. (2011). California Verbal Learning Test (California Verbal Learning Test-II). In: *Encyclopedia of Clinical Neuropsychology*. Springer International Publishing, pp.475-476.
2. Wiegner, S., & Donders, J. (1999). Performance on the California Verbal Learning Test After Traumatic Brain Injury. *Journal Of Clinical And Experimental Neuropsychology*, *21*(2), 159-170. http://dx.doi.org/10.1076/jcen.21.2.159.925
3. Elwood, R. (1995). The California Verbal Learning Test: Psychometric characteristics and clinical application. *Neuropsychology Review*, *5*(3), 173-201. http://dx.doi.org/10.1007/bf02214761
4. Baños, J., & Martin, R. (2002). California Verbal Learning Test-Second Edition. *Archives Of Clinical Neuropsychology*, *17*(5), 509-512. http://dx.doi.org/https://doi.org/10.1016/S0887-6177(01)00125-1
5. *California Verbal Learning Test® - Second Edition*. (2017). *Pearsonclinical.com*. Retrieved 1 August 2018, from http://www.pearsonclinical.com/psychology/products/100000166/california-verbal-learning-test--second-edition-cvlt-ii.html#tab-details
6. Woods, S., Delis, D., Scott, J., Kramer, J., & Holdnack, J. (2006). The California Verbal Learning Test – second edition: Test-retest reliability, practice effects, and reliable change indices for the standard and alternate forms. *Archives Of Clinical Neuropsychology*, *21*(5), 413-420. http://dx.doi.org/10.1016/j.acn.2006.06.002
7. Kim, J., & Kang, Y. (1999). Normative Study of the Korean-California Verbal Learning Test (K-CVLT). *The Clinical Neuropsychologist*, *13*(3), 365-369. http://dx.doi.org/10.1076/clin.13.3.365.1740

**THE FUNCTIONAL INDEPENDENCE MEASURE (FIM), COGNITIVE SUBSCALE (FIM-Cog)**

| Descriptive | Method of Development | Developed by ACRM/AAPMR task force to improve rehabilitation services through a software system w/ uniform measurement of outcomes, and established reliability/validity; created through literature review of other measures^1^ |
| --- | --- | --- |
|  | Purpose | 18-item measure to assess motor and cognitive dimensions in rehabilitation^1^ |
|  | Content | 6 areas of function (self-care, sphincter control, transfers, locomotion, communication, and social cognition)^1^ – motor (13 items) and cognitive (5 items – comprehension, expression, social interaction, problem solving and memory)^2,3^ |
|  | Response – Options | Actions as observed by the examiner or short answer responses by the patient^1,4^ |
|  | Recall Period | Not applicable |
|  | Endorsements | Not available online; requires UDSMR subscription^5^ |
| Practical | To obtain | Subscription information: https://www.udsmr.org/WebModules/FIM/Fim_  About.aspx^5^ User manual: https://www.va.gov/vdl/documents/Clinical/Func  _Indep_Meas/fim_user_manual.pdf^6^ |
|  | Method of Administration | Examiner observes and scores patient functioning; trained interviewers can conduct telephone interviews post-discharge^1^ |
|  | Scoring and Interpretation | - Individual items: scores range from 1 (total assistance) to 7 (complete independence); patients with scores < 6 require some level of assistance^1,3^ - Total: sum of individual, ranging from 18-126^1^ |
|  | Respondent Burden | 30-45 minutes to complete^4^ |
|  | Admin Burden | Must purchase subscription^5^ and receive certification^2^ |
|  | Translations | Afrikaans, English, Finnish, French, German, Italian, Portuguese, Spanish, Swedish, Turkish^2^ |
| Critical appraisal value | Strengths, cautions, clinical and research applicability | Worse score associated w/ increased age and presence of comorbid conditions^6^ |

FIM, Functional Independence Measure (former acronym, now simply FIM Instrument); Uniform Data System for Medical Rehabilitation

**References**

1. Wright, J. (2011). Functional Independence Measure. In: Encyclopedia of Clinical Neuropsychology. Springer International Publishing, pp.1112-1113.
2. Chan, C., & Miller, W. (2013). *Functional Independence Measure (FIM)* (p. Vancouver). SCIRE Project. Retrieved from http://scireproject.com/wp-content/uploads/Clinician-Summary-v.5.0_FIM.pdf
3. Granger, C., Hamilton, B., Linacre, J., Heinemann, A., & Wright, B. (1993). Performance Profiles of the Functional Independence Measure. *American Journal Of Physical Medicine & Rehabilitation*, *72*(2), 84-89. http://dx.doi.org/10.1097/00002060-199304000-00005
4. *Functional Independence Measure (FIM)*. (2017). *Apntoolkit.mcmaster.ca*. Retrieved 27 July 2017, from http://apntoolkit.mcmaster.ca/index.php?option=com_content&view=article&id=301:functional-independence-measure-fim&Itemid=58
5. *About the FIM System*. (n.d.). *Udsmr.org*. Retrieved 27 July 2018, from https://www.udsmr.org/WebModules/FIM/Fim_About.aspx
6. Dodds, T., Martin, D., Stolov, W., & Deyo, R. (1993). A validation of the Functional Independence Measurement and its performance among rehabilitation inpatients. *Archives Of Physical Medicine And Rehabilitation*, *74*(5), 531-536. http://dx.doi.org/10.1016/0003-9993(93)90119-u

**THE MINI-MENTAL STATE EXAMINATION (MMSE)**

| Descriptive | Method of Development | Initially developed to assess cognitive deficits within dementia, affective disorder, schizophrenia, personality disorder w/ drug abuse, and neuroses; developed w/ a focus on cognitive aspects of mental functions rather than mood, abnormal mental experiences or thought^1^ |
| --- | --- | --- |
|  | Purpose | To assess mental status and track cognitive impairment/recovery^1-2^ |
|  | Content | 11 questions covering 5 domains of cognition: orientation to time and place, registration (object memory), attention and calculation, recall (short-term), language (object naming, word repetition, comprehension, reading, writing, and drawing)^1-2^ |
|  | Response - Options | Orientation, registration, attention and calculation, and recall require verbal responses; language requires verbal and written responses^2^ |
|  | Recall Period | Within 5^1-2^-15^3^ minutes (total test time): recall domain requires short-term recall of 3 words learned in registration domain^2^ |
|  | Endorsements | Questions publicly viewable,^2^ forms and scoring kits available to individuals w/ appropriate qualification^3^ |
| Practical | To obtain | Questions can be viewed at: https://www.mountsinai.on.ca/care/psych/on-call-resources/on-call-resources/mmse.pdf^2^  Forms/manual can be purchased from PAR: https://www.parinc.com/Products/Pkey/237^3^ |
|  | Method of Administration | Orientation: patient is asked year/season/date/day/month, and state/country/town/hospital/floor^2^  Registration: examiner names 3 objects and patient is asked to repeat, multiple trials used until all 3 words are learned^2^  Attention and calculation: patient is asked to carry out 5 serial additions of 7, or spell a word backwards^2^  Recall: patient is asked to name the 3 objects from the registration domain^2^  Language: patient is asked to name 2 objects, repeat a series of words, follow a 3-stage command, read and obey an instructive phrase, write a sentence, and copy a design^2^ |
|  | Scoring and Interpretation | Maximum score is out of 30: orientation (10), registration (3), attention and calculation (5), recall (3), and language (9)^1-2^  Recommended score categories: normal cognitive functioning (27–30), mild cognitive impairment (21–26), moderate cognitive impairment (11–20), severe cognitive impairment (0–10) reflecting severe cognitive impairment;^1^ score of ≤23 typically interpreted as cognitive impairment^2^ |
|  | Respondent Burden | 5^1-2^-15^3^ minutes to complete |
|  | Admin Burden | 5^1-2^-15^3^ minutes to administer + 5 minutes to score; Level S qualification required for purchase of materials^3^ |
|  | Translations | Available in >70 languages^3^ |
| Critical appraisal value | Strengths, cautions, clinical and research applicability | Quickly administered and suitable for routine use^2^  Can measure change in mental status w/ repeated use^2^  Sex not significantly associated with score ^4^  Less practical for patients w/ physical impairments that impede participation intubated or those w/ low English literacy^2^  Less sensitive than MoCA at detecting TBI, ^5^ post-stroke cognitive impairment^6^ and dementia^7^ Fewer years of education related to decreased specificity and increased sensitivity; ^8^ educational background observed to have a strong effect on writing, reading and obeying, and copy design ^9^  Higher age related to decreased specificity ^9^ |

MoCA, Montreal Cognitive Assessment; TBI, traumatic brain injury

**References**

1. Schatz, P. (2011). Mini-Mental State Exam. In: *Encyclopedia of Clinical Neuropsychology*. Springer International Publishing, pp.1627-1629.
2. Kurlowicz, L. and Wallace, M. (1999). *The Mini Mental State Examination (MMSE)*. The Hartford Institute for Geriatric Nursing, Division of Nursing, New York University, pp.1-2. Retrieved from: https://www.mountsinai.on.ca/care/psych/on-call-resources/on-call-resources/mmse.pdf
3. *MMSE (Mini-Mental State Examination)*. (2018). *www.parinc.com*. Retrieved 30 July 2018, from https://www.parinc.com/Products/Pkey/237
4. Magni, E., Binetti, G., Padovani, A., Cappa, S.F., Bianchetti, A., & Trabucchi, M. (1996). The Mini-Mental State Examination in Alzheimer's disease and multi-infarct dementia. *International Psychogeriatrics*, *8*(1), 127-134.
5. Zhang, H., Zhang, X.N., Zhang, H.L., Huang, L., Chi, Q., Zhang, X., & Yun, X. (2016). Differences in cognitive profiles between traumatic brain injury and stroke: A comparison of the Montreal Cognitive Assessment and Mini-Mental State Examination. *Chinese Journal of Traumatology*, *19*, 271-274.
6. Fu, C., Jin, X., Chen, B., Xue, F., Niu, H., Guo, R., … & Zhang, Y. Comparison of the Mini‐Mental State Examination and Montreal Cognitive Assessment executive subtests in detecting post‐stroke cognitive impairment. *Geriatrics and Gerontology International*, *17*(12), 2329-2335. http://dx.doi.org/10.1111/ggi.13069
7. Dong, Y., Lee, W.Y., Basri, N.A., Collinson, S.L., Merchant, R.A. Venketasubramanian, N., & Chen, C.L. (2012). The Montreal Cognitive Assessment is superior to the Mini–Mental State Examination in detecting patients at higher risk of dementia. *International Psychogeriatrics*, *24*(11), 1749-1755. http://dx.doi.org/10.1017/S1041610212001068
8. Tombaugh, T.N., McDowell, I., Kristjansson, B., & Hubley, A.M. (1996). Mini-Mental State Examination (MMSE) and the Modified MMSE (3MS): A Psychometric Comparison and Normative Data. *Psychological Assessment*, *8*(1), 48-59. http://dx.doi.org/10.1037/1040-3590.8.1.48
9. Shyu, Y.I., & Yip, P.K. (2001). Factor structure and explanatory variables of the Mini-Mental State Examination (MMSE) for elderly persons in Taiwan. *Journal of the Formosan Medical Association*, *100*(10),676-683.

**THE HOPKINS VERBAL LEARNING TEST (HVLT)**

| Descriptive | Method of Development | Developed to provide brief method of longitudinal assessment; semantic categories chosen from previously determined set (4 most common words in each, w/ 2 most common designated distractors)^1-3^ |
| --- | --- | --- |
|  | Purpose | To evaluate memory and verbal learning^1^ |
|  | Content | Assesses immediate and delayed recall, and delayed recognition^2^  Consists of a 12-word list (4 words each from 3 semantic categories) w/ 6 alternate forms, a 24-word recognition list (12 target words, 6 semantically-related and 6 semantically-unrelated distractor words)^1,3^ |
|  | Response – options | Patient must learn a list of words and demonstrate recall/recognition^1^ |
|  | Recall period | Not applicable |
|  | Endorsements | Available to individuals w/ appropriate qualification^2^ |
| Practical | To obtain | HVLT-R available from PAR: http://www4.parinc.com/Products/Product.aspx?ProductID=HVLT-R^2^ |
|  | Method of administration | HVLT: patient is verbally presented w/ 12 words (2 words/s) and immediate free recall is assessed; repeated 2x (total 3 trials) and followed by recognition^3^  HVLT-R: DR and recognition assessed ~25 minutes later^4^ |
|  | Scoring and interpretation | HVLT: Trials 1/2/3 # recalled, Learning (higher of Trials 2/3 – Trial 1), TR (3-trial total), Recognition TPs/FPs, Recognition Discrim. (TP – FP), and RB  HVLT-R: Trial 4 (DR) # recalled, % retained (higher of Trials 2/3 divided by Trial 4)^4^ |
|  | Respondent burden | HVLT-R: 5-10 minutes testing time + 25 minutes delay^2^ |
|  | Admin burden | Requires Level C qualification; must purchase; 2 minutes to score^2^ |
|  | Translations | English^2^, French^5^, Chinese^6^ |
| Critical Appraisal Value | Strengths, Cautions, Clinical and Research Applicability | Use of 6 alternate forms is practical in patient populations that require follow-up evaluation and assessment of treatment gains^1^ |
|  |  | HVLT: weakly correlated w/ level of education (r = 0.27; p = 0.04) ^7^  HVLT-R: age seen to be significant predictor (β = -0.28; p = 0.002) ^8^ |
|  |  | HVLT: better sensitivity than MMSE in detecting dementia patients ^7^  HVLT-R: better sensitivity/specificity than MMSE and CogState in detecting MCI patients ^8^ |

DR, delayed recall; FP, false positive; HVLT/HVLT-R, Hopkins Verbal Learning Test/Hopkins Verbal Learning Test-Revised; MCI; mild cognitive impairment; MMSE, Mini–Mental State Examination; RB, response bias; SD, standard deviation; TP, true positive; TR, total recall

**References**

1. Belkonen, S. (2011). Hopkins Verbal Learning Test. In: *Encyclopedia of Clinical Neuropsychology*. Springer International Publishing, pp.1264-1265.
2. *HVLT-R (Hopkins Verbal Learning Test-Revised)*. (2017). *www4.parinc.com*. Retrieved 1 August 2017, from http://www4.parinc.com/Products/Product.aspx?ProductID=HVLT-R
3. Brandt, J. (1991). The Hopkins verbal learning test: Development of a new memory test with six equivalent forms. *Clinical Neuropsychologist*, *5*(2), 125-142. http://dx.doi.org/10.1080/13854049108403297
4. Benedict, R., Schretlen, D., Groninger, L., & Brandt, J. (1998). Hopkins Verbal Learning Test - Revised: Normative Data and Analysis of Inter-Form and Test-Retest Reliability. *The Clinical Neuropsychologist (Neuropsychology, Development And Cognition: Section D)*, *12*(1), 43-55. http://dx.doi.org/10.1076/clin.12.1.43.1726
5. Rieu, D., Bachoud-Lévi, A., Laurent, A., Jurion, E., & Dalla Barba, G. (2006). Adaptation française du « Hopkins verbal learning test ». *Revue Neurologique*, *162*(6-7), 721-728. http://dx.doi.org/10.1016/s0035-3787(06)75069-x
6. Shi, J., Tian, J., Wei, M., Miao, Y., & Wang, Y. (2012). The utility of Hopkins verbal learning test (Chinese version) for screening dementia and mild cognitive impairment in a Chinese population. *Alzheimer's & Dementia*, *8*(4), P360-P361. http://dx.doi.org/10.1016/j.jalz.2012.05.988
7. Frank, R., & Byrne, G. (2000). The clinical utility of the Hopkins Verbal Learning Test as a screening test for mild dementia. *International Journal Of Geriatric Psychiatry*, *15*(4), 317-324. http://dx.doi.org/10.1002/(sici)1099-1166(200004)15:4<317::aid-gps116>3.3.co;2-z
8. de Jager, C., Schrijnemaekers, A., Honey, T., & Budge, M. (2009). Detection of MCI in the clinic: evaluation of the sensitivity and specificity of a computerised test battery, the Hopkins Verbal Learning Test and the MMSE. *Age And Ageing*, *38*(4), 455-460. http://dx.doi.org/10.1093/ageing/afp068

**THE IMMEDIATE POST-CONCUSSION ASSESSMENT AND COGNITIVE TESTING (IMPACT)**

| Descriptive | Method of Development | Developed to have an inexpensive and easily-administered instrument; alternative to time-consuming and expensive paper and pencil methods w/ poor norms, sensitivity, specificity and vulnerability to practice effects^1,2^ |
| --- | --- | --- |
|  | Purpose | To evaluate baseline and post-concussion cognitive performance^1^ |
|  | Content | WM: assesses immediate/learning and delayed/retention verbal recognition memory  DM (version 2.0): assesses immediate/learning and delayed/retention spatial recognition memory  X’s and O’s: assesses visual working memory and cognitive speed  SM: assesses visual-motor speed and memory  CM: assesses visual-motor speed and impulse inhibition  TLM: assesses verbal working memory and cognitive speed^2^ |
|  | Response – options | Responses to each module made using a computer and mouse^1^ |
|  | Recall period | Not applicable |
|  | Endorsements | Available to individuals w/ appropriate qualification^1^ |
| Practical | To obtain | Available from ImPACT Applications: https://www.impacttest.com/purchase/form^3^ |
|  | Method of administration | Patient must complete each online module, demographic information and PCSS^1^; details about specific modules not reported |
|  | Scoring and interpretation | VEM: composite of learning and delayed WM, SM, and TLM scores  VIM: learning and delayed DM, X’s and O’s % correct  VMPS: X’s and O’s avg. correct distractors, SM avg. correct responses, Three Letters Memory # correct responses  RT: X’s and O’s avg. correct RT, SM avg. weighted corrected RT, CM avg. correct RT  IC: X’s and O’s #incorrect distractors, CM # errors^2^ |
|  | Respondent burden | 25 minutes to complete^1^ |
|  | Admin burden | Requires appropriate certification, computer access^1^, and test package and training fees^3^; scoring and data storage automatically completed by software^1^ |
|  | Translations | English |
| Critical Appraisal Value | Strengths, Cautions, Clinical and Research Applicability | Can be administered with little supervision^4^ |
|  |  | BMI was a predictor of VIM score (∆R^2^ = 0.02; p = 0.03)^5^ |

avg. average; BMI, Body Mass Index; CM; Colour Match; CPT, Continuous Performance Test; DM, Design Memory; IC, Impulse Control; ImPACT, Immediate Post Concussion Assessment and Cognitive Testing; RT, Reaction Time; SM, Symbol Match; TLM, Three Letter Memory; VEM, Verbal Memory; VF, Verbal Fluency; VIM, Visual Memory; VMPS, Visual-Motor Processing Speed; WM, Word Memory

**References**

1. *The ImPACT Test*. (2017). *www.impacttest.com*. Retrieved 1 August 2018, from https://www.impacttest.com/products/?ImPACT-Immediate-Post-Concussion-Assessment-and-Cognitive-Test-2
2. Schatz, P., Pardini, J., Lovell, M., Collins, M., & Podell, K. (2006). Sensitivity and specificity of the ImPACT Test Battery for concussion in athletes. *Archives Of Clinical Neuropsychology*, *21*(1), 91-99. http://dx.doi.org/10.1016/j.acn.2005.08.001
3. *Purchase ImPACT*. (2017). *www.impacttest.com*. Retrieved 1 August 2017, from https://www.impacttest.com/purchase/form
4. Maroon, J., Lovell, M., Norwig, J., Podell, K., Powell, J., & Hartl, R. (2000). Cerebral Concussion in Athletes: Evaluation and Neuropsychological Testing. *Neurosurgery*, *47*(3), 659-672. http://dx.doi.org/10.1097/00006123-200009000-00027
5. Fedor, A., & Gunstad, J. (2013). Higher BMI Is Associated with Reduced Cognitive Performance in Division I Athletes. *Obesity Facts*, *6*(2), 185-192. http://dx.doi.org/10.1159/000351138

**THE PACED AUDITORY SERIAL ADDITION TEST (PASAT)**

| Descriptive | Method of Development | Initially developed to assess immediate memory and attention using visual stimuli, and later adapted for use in a TBI population^1^ |
| --- | --- | --- |
|  | Purpose | To evaluate rate of information processing in TBI and MS patients^1^ |
|  | Content | Sustained/divided attention, concentration, information processing speed^2^ |
|  | Response – options | Patient is presented with a series of single digits; must add last presented digit to previous one and state sum^1,3^ |
|  | Recall period | Not applicable |
|  | Endorsements | Available to individuals w/ appropriate qualification^4^ |
| Practical | To obtain | Available from: http://pasat.us/^4^  Manual: http://www.nationalmssociety.org/NationalMSSociety/media/  MSNationalFiles/Brochures/10-2-3-31-MSFC_Manual_and_Forms.pdf^5^ |
|  | Method of administration | A series of 61digits is played in 4 trials (same series presented at 4 ISIs – 2.4, 2.0, 1.6, and 1.2 s^1,3^– w/ a 60 s break b/w trials)^3^; each is digit presented for 0.4 s^1,3^; 1.6 and 1.2 s trials are administered if score is ≥20 on 2.0s trial or ≥40s on 2.4 trial^3^; multiple versions where ISI or length of trial vary^3^ |
|  | Scoring and interpretation | Total score: total # correct in each trial (max = 60)^1,3^  Composite score: total # correct summed over all trials^1,3^  Avg. time/correct response: ratio of # correct to trial duration (ISI x 60)^1,3^  Other: # times stimulus added to previous answer, longest string correct, RT^3^ |
|  | Respondent burden | 6-8 to 15-20 minutes to complete, depending on version used^3^ |
|  | Admin burden | Requires training^3^; must purchase forms, recordings, and CD player ^4^ |
|  | Translations | Available in 27 languages^4^; computerized versions available^3^ |
| Critical Appraisal Value | Strengths, Cautions, Clinical and Research Applicability | High internal consistency and test-retest reliability in non-TBI populations, but need to be documented in TBI patients^2, 6-7,8-11^ |
|  |  | Low divergent validity – performance appears to be related to cognitive abilities other than attention and processing speed^2^  Low score cannot be used to determine presence of cognitive dysfunction^1^  Documented practice effects, particularly at shorter ISI values^1^ |
|  |  | Worse score associated w/ increased age, decreased IQ, and lower math ability^1^ |

avg, average; ISI, interstimulus interval; IQ, intelligence quotient; MS, multiple sclerosis; TBI, traumatic brain injury;

**References**

1. Tombaugh, T. (2006). A comprehensive review of the Paced Auditory Serial Addition Test (PASAT). *Archives of Clinical Neuropsychology*, 21(1), pp.53-76.
2. Sherman, E., Strauss, E. and Spellacy, F. (1997). Validity of the paced auditory serial addition test (pasat) in adults referred for neuropsychological assessment after head injury. *The Clinical Neuropsychologist*, 11(1), pp.34-45. http://dx.doi.org/10.1080/13854049708407027
3. Correia, S. (2011). Paced Auditory Serial Attention Test. In: *Encyclopedia of Clinical Neuropsychology*. Springer International Publishing, pp.1840-1844.
4. *PASAT form*. (n.d.). *Pasat.us*. Retrieved 19 July 2017, from http://www.pasat.us/
5. Fischer, J., Jak, A., Kniker, J., Rudick, R. and Cutter, G. (2001). *Multiple Sclerosis Functional Composite: Administration and Scoring Manual*. National Multiple Sclerosis Society, pp.14-18.
6. Egan, V. (1988). PASAT: Observed correlations with IQ. *Personality and Individual Differences*, 9(1), pp.179-180. http://dx.doi.org/10.1016/0191-8869(88)90046-3
7. Crawford, J., Obonsawin, M. and Allan, K. (1998). PASAT and Components of WAIS-R Performance: Convergent and Discriminant Validity. *Neuropsychological Rehabilitation*, 8(3), pp.255-272. http://dx.doi.org/10.1080/713755575
8. McCaffrey, R., Cousins, J., Westervelt, H., Martynowicz, M., Remick, S., Szebenyi, S., Wagle, W., Bottomley, P., Hardy, C. and Haase, R. (1995). Practice effects with the NIMH AIDS abbreviated neuropsychological battery. *Archives of Clinical Neuropsychology*, 10(3), pp.241-250. https://doi.org/10.1016/0887-6177(94)00048-U
9. McCaffrey, R., Westervelt, H. and Haase, R. (2001). Serial neuropsychological assessment with the National Institute of Mental Health (NIMH) AIDS Abbreviated Neuropsychological Battery. *Archives of Clinical Neuropsychology*, 16(1), pp.9-18. https://doi.org/10.1016/S0887-6177(99)00055-4
10. Sjøgren, P., Thomsen, A. and Olsen, A. (2000). Impaired Neuropsychological Performance in Chronic Nonmalignant Pain Patients Receiving Long-Term Oral Opioid Therapy. *Journal of Pain and Symptom Management*, 19(2), pp.100-108. https://doi.org/10.1016/S0885-3924(99)00143-8
11. Beglinger, L., Gaydos, B., Tangphaodaniels, O., Duff, K., Kareken, D., Crawford, J., Fastenau, P. and Siemers, E. (2005). Practice effects and the use of alternate forms in serial neuropsychological testing. Archives of Clinical Neuropsychology, 20(4), pp.517-529. https://doi.org/10.1016/j.acn.2004.12.003

**THE REY AUDITORY VERBAL LEARNING TEST (RAVLT)**

| Descriptive | Method of Development | Developed in French in 1941 and adapted to English in 1959; designed to provide different measures that correspond to different memory processes^1^ |
| --- | --- | --- |
|  | Purpose | To evaluate nature/ severity of memory dysfunction and changes in memory^1^ |
|  | Content | Assesses inhibition, retention, encoding, retrieval, and organization^2^  Contains 2 15-item lists of nouns (List A and interference List B) + 50-item recognition list w/ 30 A/B words and 20 distractor words^1^ |
|  | Response – options | Free recall (FR): patient must recall as many A/B words as possible; can be short-delay/immediate (SDFR) or long-delay/after 20 minutes (LDFR)  Recognition: patient must classify recognition words as targets or distractors^1^ |
|  | Recall period | Not applicable |
|  | Endorsements | Available to individuals w/ appropriate qualification^2^ |
| Practical | To obtain | Available from PAR: http://www4.parinc.com/Products/Product.aspx?ProductID=RAVLT^2^ |
|  | Method of administration | Each list is verbally presented; 5 List A trials and 1 List B trial w/ immediate FR after each; List A SDFR assessed immediately after List B presentation; List A LDFR assessed 20 minutes later; recognition list assessed at the end^1^ |
|  | Scoring and interpretation | List A Trials 1/2/3/4/5/1-5/SDFR/LDFR and List B: # correctly recalled^1,3^  Recognition: hits and false positives^4^  PI: List B/Trial 1  RI: SDFR/Trial 5^3^ |
|  | Respondent burden | 10-15 minutes to complete^2^ |
|  | Admin burden | Requires Level C qualification; must purchase test kit^2^ |
|  | Translations | English^2^, French, Spanish, Greek, Brazilian Portuguese, Hebrew^1^ |
| Critical Appraisal Value | Strengths, Cautions, Clinical and Research Applicability | Several alternate forms in English version to diminish practice effects^1^ |
|  |  | Low to moderate effect of age (r = –0.22 to –0.55) and education (r = –0.14 to –0.54)^5^ |

FR, free recall; LDFR, long delay free recall; MCI; mild cognitive impairment; NS, non-significant/non-significantly; PAR, Psychological Assessment Resources; RAVLT, Rey Auditory Verbal Learning Test; SDFR, short delay free recall

**References**

1. Bean, J. (2011). Rey Auditory Verbal Learning Test, Rey AVLT. In: *Encyclopedia of Clinical Neuropsychology*. Springer International Publishing, pp.2174-2175.
2. *RAVLT (Rey Auditory Verbal Learning Test: A Handbook)*. (2012). *www4.parinc.com*. Retrieved 22 August 2017, from http://www4.parinc.com/Products/Product.aspx?ProductID=RAVLT
3. de Sousa Magalhães, S., Fernandes Malloy-Diniz, L., & Cavalheiro Hamdan, A. (2017). Validity Convergent and Reliability Test-Retest of the Rey Auditory Verbal Learning Test. *Clinical Neuropsychiatry*, *9*(3), 129-137.
4. Geffen, G., Butterworth, P., & Geffen, L. (1994). Test-retest reliability of a new form of the auditory verbal learning test (AVLT). *Archives Of Clinical Neuropsychology*, *9*(4), 303-316. http://dx.doi.org/10.1093/arclin/9.4.303
5. Delaney, R., Prevey, M., Cramer, J., Mattson, R., & VA Epilepsy Cooperative Study #264 Research Group. (1992). Test-retest comparability and control subject data for the rey-auditory verbal learning test and rey-osterrieth/taylor complex figures. *Archives Of Clinical Neuropsychology*, *7*(6), 523-528. http://dx.doi.org/10.1093/arclin/7.6.523

**THE REY OSTERRIETH COMPLEX FIGURE TEST (ROCF)**

| Descriptive | Method of Development | Developed by Rey in 1941; 36-point scoring system developed and normed by Osterrieth in 1944, and by Meyers and Meyers in 1995 (most commonly used)^1^ |
| --- | --- | --- |
|  | Purpose | To assess memory and organization in patients (6-89 years) w/ brain damage^1,2^ |
|  | Content | Assesses visuospatial perception, visual memory, long-term memory encoding/storage/retrieval, strategic planning and organization, attention, concentration, fine-motor coordination^3^ |
|  | Response – Options | Patient is presented w/ a figure and must either copy, draw or recognize aspects of it from memory^1^ |
|  | Recall Period | Not applicable |
|  | Endorsements | Available to individuals w/ appropriate qualification^2^ |
| Practical | To obtain | Meyers and Meyers version (RCFT) available from PAR: http://www4.parinc.com/Products/Product.aspx?ProductID=RCFT^2^ |
|  | Method of Administration | Patient must copy a complex geometric figure (Copy Trial), followed by immediate and/or delayed reproduction from memory (IR/DR Trials); delay timing of the DR Trial can vary^3^; patient must complete recall trials w/i 10 minute time limit; RCFT ends w/ a Recognition Trial where patients are given 12 original design features + 12 foils and must circle original details; sequence of strokes can be tracked by pen-switching (providing a different coloured pen when specific points on the figure are reached) or following w/ a flow chart^1^ |
|  | Scoring and Interpretation | - Figure divided into 18 units w/ a score of 0 (inaccurately drawn/ unrecognizable/omitted + incorrectly placed), 0.5, 1, or 2 (accurately drawn + correctly placed); unit scores are summed to give a total ranging from 0 to 36; low Copy score corresponds to reduced visual-perceptual and visuomotor skills; low Recall scores correspond to reduced visuospatial recall ability; differences in IR and DR indicate disruptions in encoding, storage and retrieval processes^3^ |
|  | Respondent Burden | Overall 40-60 minutes to complete (10 minutes each for Copy, IR and DR + 20-30 minute delay)^3^ |
|  | Admin Burden | Requires Level C qualification; must purchase test kit; 15 minutes to score^2^ |
|  | Translations | English^2^ |
| Critical appraisal value | Strengths, cautions, clinical and research applicability | COWAT, TMT B and WCST # perseverative responses were significant predictors of Copy, IR, DR and recognition (R^2^ = 0.11-0.16; p < 0.05)^4^  Age had a significant effect on Copy, IR and DR scores (F = 5.44-8.32; p < 0.001) w/ older individuals scoring significantly lower (p < 0.05)^5^  Sex had a significant effect on Copy, IR and DR scores (F = 4.76-23.51; p < 0.05) w/ males producing more accurate copies and recalling more information^5^  IQ had a significant effect on Copy, IR and DR scores (F = 4.35-7.50; p < 0.05) where individuals w/ IQ scored higher than those with lower IQ^19d^  NS differences in Copy, IR and DR scores b/w pen-switching and flow chart methods (t = 0.14, –0.31 - –0.19; p ≥ 0.05) using the 36-point scoring system^6^ |

COWAT, Controlled Oral Word Association Test; DR, Delayed Recall; GPT, Grooved Pegboard Test; IQ, Intelligence Quotient; IR, Immediate Recall; RCFT, Rey Complex Figure Test and Recognition Trial; ROCF, Rey–Osterrieth Complex Figure Test; TMT, Trail Making Test; US, United states; VPA, Verbal Paired Associates; Vocab, Vocabulary; VR, Visual Reproduction; WCST, Wisconsin Card Sorting Test

**References**

1. Jerskey, B., & Meyers, E. (2011). Rey Complex Figure Test. In: *Encyclopedia of Clinical Neuropsychology*. Springer International Publishing, pp.2176-2178.
2. *RCFT (Rey Complex Figure Test and Recognition Trial)*. (2012). *www4.parinc.com*. Retrieved 22 August 2017, from http://www4.parinc.com/Products/Product.aspx?ProductID=RCFT
3. Shin, M., Park, S., Park, S., Seol, S., & Kwon, J. (2006). Clinical and empirical applications of the Rey–Osterrieth Complex Figure Test. *Nature Protocols*, *1*(2), 892-899. http://dx.doi.org/10.1038/nprot.2006.115
4. Schwarz, L., Penna, S., & Novack, T. (2009). Factors contributing to performance on the Rey Complex Figure Test in individuals with traumatic brain injury. *The Clinical Neuropsychologist*, *23*(2), 255-267. http://dx.doi.org/10.1080/13854040802220034
5. Gallagher, C., & Burke, T. (2007). Age, gender and IQ effects on the Rey-Osterrieth Complex Figure Test. *British Journal Of Clinical Psychology*, *46*(1), 35-45. http://dx.doi.org/10.1348/014466506x106047
6. Ruffolo, J., Javorsky, D., Tremont, G., Westervelt, H., & Stern, R. (2001). A comparison of administration procedures for the Rey-Osterrieth Complex Figure: Flowcharts versus pen switching. *Psychological Assessment*, *13*(3), 299-305. http://dx.doi.org/10.1037//1040-3590.13.3.299

**THE SYMBOL DIGIT MODALITIES TEST (SDMT)**

| Descriptive | Method of Development | Developed by Smith in 1982; similar to WAIS DST (where presented numbers are substituted w/ corresponding symbols), but advantageous in that patients are more familiar w/ writing out numbers and are able to respond orally^1,6^ |
| --- | --- | --- |
|  | Purpose | Brief test to screen for organic cerebral dysfunction in individuals aged 8 and up^1^ |
|  | Content | Assesses attention, visual scanning, motor speed^2^, graphomotor skills, visuospatial ability, incidental memory^3^ |
|  | Response – Options | Patient must indicate a specific number (written or oral) in response to various presented geometric figures^1^ |
|  | Recall Period | Not applicable |
|  | Endorsements | Available to individuals w/ appropriate qualification^1^ |
| Practical | To obtain | Available from WPS: https://www.wpspublish.com/store/p/2955/sdmt-symbol-digit-modalities-test^1^ |
|  | Method of Administration | Patient is presented w/ a key and a series of 9 geometric figures in random order, and must pair each one w/ its corresponding number (1-9) w/i a 90s time limit^2^; can conduct written version, followed by oral; can be individual or group^1^ |
|  | Scoring and Interpretation | Possible variables include total correct^4^ and number of figures attempted^5^ |
|  | Respondent Burden | < 5 minutes to complete^1^ |
|  | Admin Burden | Requires Level C qualification; must purchase test kit and stopwatch^1^ |
|  | Translations | English, Dutch, Spanish^1^ |
| Critical appraisal value | Strengths, cautions, clinical and research applicability | Oral/written versions can accommodate patients with motor/speech disabilities^1^  Can be used w/ patients who do not speak English^1,6^  Used to assess MS patients as part of the MSFC^4^ |
|  |  | Low to moderate correlations w/ age, sex and education (r = 0.20-0.24, –0.42; p < 0.01) ^7^  Brain lesion burden and atrophy observed to be significant predictors of score, especially third ventricle width (R^2^ = 0.57; p < 0.001) ^8^  Oral/written show NS differences in age, sex and income effects^2^  Low correlation w/ gender and education (r = 0.17-0.22; p < 0.05); weak, NS correlation w/ age (r = 0.03; p ≥ 0.05) ^9^ |

DST, Digit Symbol Test; NS, non-significant/non-significantly; SDMT, Symbol Digit Modalities Test; WAIS, Wechsler Adult Intelligence Scale

**References**

1. *(SDMT) Symbol Digit Modalities Test | WPS*. (2017). *wpspublish.com*. Retrieved 22 August 2018, from https://www.wpspublish.com/store/p/2955/sdmt-symbol-digit-modalities-test
2. Sheridan, L., Fitzgerald, H., Adams, K., Nigg, J., Martel, M., & Puttler, L. et al. (2006). Normative Symbol Digit Modalities Test performance in a community-based sample. *Archives Of Clinical Neuropsychology*, *21*(1), 23-28. http://dx.doi.org/10.1016/j.acn.2005.07.003
3. Bettcher, B., Libon, D., Kaplan, E., Swenson, R., & Penney, D. (2011). Digit Symbol Substitution Test. In: *Encyclopedia of Clinical Neuropsychology*. Springer International Publishing, pp.849-852.
4. Drake, A., Weinstock-Guttman, B., Morrow, S., Hojnacki, D., Munschauer, F., & Benedict, R. (2009). Psychometrics and normative data for the Multiple Sclerosis Functional Composite: replacing the PASAT with the Symbol Digit Modalities Test. *Multiple Sclerosis Journal*, *16*(2), 228-237. http://dx.doi.org/10.1177/1352458509354552
5. McCaffrey, R., Krahula, M., Heimberg, R., Keller, K., & Purcell, M. (1988). A comparison of the trail making test, symbol digit modalities test, and the Hooper visual organization test in an inpatient substance abuse population. *Archives Of Clinical Neuropsychology*, *3*(2), 181-187. http://dx.doi.org/10.1093/arclin/3.2.181
6. Benedict, R., Smerbeck, A., Parikh, R., Rodgers, J., Cadavid, D., & Erlanger, D. (2012). Reliability and equivalence of alternate forms for the Symbol Digit Modalities Test: implications for multiple sclerosis clinical trials. *Multiple Sclerosis Journal*, *18*(9), 1320-1325. http://dx.doi.org/10.1177/1352458511435717
7. Berrigan, L., Fisk, J., Walker, L., Wojtowicz, M., Rees, L., Freedman, M., & Marrie, R. (2014). Reliability of Regression-Based Normative Data for the Oral Symbol Digit Modalities Test: An Evaluation of Demographic Influences, Construct Validity, and Impairment Classification Rates in Multiple Sclerosis Samples. *The Clinical Neuropsychologist*, *28*(2), 281-299. http://dx.doi.org/10.1080/13854046.2013.871337
8. Benedict, R., Duquin, J., Jurgensen, S., Rudick, R., Feitcher, J., & Munschauer, F. et al. (2008). Repeated assessment of neuropsychological deficits in multiple sclerosis using the Symbol Digit Modalities Test and the MS Neuropsychological Screening Questionnaire. Multiple Sclerosis Journal, 14(7), 940-946. http://dx.doi.org/10.1177/1352458508090923
9. Crowe, S., Benedict, T., Enrico, J., Mancuso, N., Matthews, C., & Wallace, J. (1999). Cognitive Determinants of Performance on the Digit Symbol-Coding Test, and the Symbol Search Test of the Wais-III, and the Symbol Digit Modalities Test: An Analysis in a Healthy Sample. *Australian Psychologist*, *34*(3), 204-210. http://dx.doi.org/10.1080/00050069908257455

**THE STROOP TASK**

| Descriptive | Method of Development | Developed in 1929 by Jaensch; English version introduced in 1935 by Stroop; multiple versions w/ 100 items presented in either 10x10 or 5x20 girds^1^; current standardized version uses a 5x20 grid and was introduced by Golden in 1978 and updated in 2002^2^ |
| --- | --- | --- |
|  | Purpose | 3 subtask measure developed to study interference in serial verbal reactions^3^; used to distinguish normal, brain-damaged, and non-brain-damaged psychiatric patients of ages 15 to 90^4^ |
|  | Content | Assesses cognitive flexibility, resistance to interference, cognitive stress coping, and ability to process complex input^4^  Measures interference of colour stimuli on reading words and of word stimuli on naming colour^1^  Standardized version consists of 3 cards:  W – 100 colour names (red, green, and blue) printed in black, and placed randomly on a white background  C – 100 coloured bars (XXXX) printed in red, green or blue, and placed randomly on a white background  Incongruent CW – 100 colour names printed in red, green or blue such that no word matches the colour it names^5-6^ |
|  | Response -Options | W – RCNb  C – NC  CW – RCNd or NCWd^3^ |
|  | Recall Period | Not applicable |
|  | Endorsements | Available to individuals w/ appropriate qualifications^4^ |
| Practical | To obtain | Complete test kit available from PAR: http://www4.parinc.com/Products/ Product.aspx?ProductID=STROOP#Items^2^ |
|  | Method of Administration | Can be administered individually or as part of a group; 3 8.5” x 11” cards presented in W, C, CW order; must go down the columns while reading words or naming colours; given 45 seconds for each task, and must circle last completed item; if < 45 seconds, patient must begin list again^4^ |
|  | Scoring and Interpretation | Basic scores: W, C, and CW # correct responses, and Interference Score^3^ (difference b/w W/C and CW^3,5^: RCNb – RCNd interference of colour on reading; NC – NCWd interference of words on naming colours)^3^  Derived scores: error scores and patterns of responses (serial scores)^7^  Normative scores for current version based on normal sample of 300 individuals, aged 15-90, w/ 2-20 year education range; ideal scores predicted based on age and education; difference b/w obtained and predicted scores is converted to a T score^5^ |
|  | Respondent Burden | 5 minutes to complete^4^ |
|  | Admin Burden | Requires Level C qualification; must purchase test pages and stopwatch^2^ |
|  | Translations | English, Danish, Dutch, French, German, Greek, Spanish^8^ |
| Critical appraisal value | Strengths, cautions, clinical and research applicability | Validated in TBI populations  Short administration time, relatively inexpensive^4^ |
|  |  | Reliability not consistently determined in neurologic populations; high practice effects |
|  |  | W, C and CW can distinguish b/w frontal and non-frontal patients – W (d = –0.45 - –0.33; p < 0.05); CW can distinguish b/w left and right frontal patients (d = -0.39; p < 0.05)^9^  Observed sex differences – females consistently seen to be better at colour naming than males |

CW, Colour Word; NC, name colours; NCWd, name colour of word; NS, non-significant/non-significantly; RCNb, read colour names in black; RCNd, read colour name printed in a different color; RT, reaction/response time; TBI, traumatic brain injury; W, Word

**References**

1. Rozenblatt, Shahal. (2011). Stroop Color Word Test. In: Encyclopedia of Clinical Neuropsychology. Springer International Publishing, pp.2505-2405.
2. Golden, C. *Stroop Color and Word Test* (pp. 751-758). Chicago: Stoelting Company. Retrieved from http://www.killianphd.com/portals/0/stroop%20color%20word%20interference%20test.pdf
3. Stroop, J. (1992). Studies of interference in serial verbal reactions. *Journal Of Experimental Psychology: General*, *121*(1), 15-23. http://dx.doi.org/10.1037//0096-3445.121.1.15
4. *Stroop Color and Word Test*. (2012). *www4.parinc.com*. Retrieved 1 August 2017, from http://www4.parinc.com/Products/Product.aspx?ProductID=STROOP#Items
5. Jensen, A., & Rohwer, W. (1966). The stroop color-word test: A review. *Acta Psychologica*, *25*, 36-93. http://dx.doi.org/https://doi.org/10.1016/0001-6918(66)90004-7
6. Moller, J., Cluitmans, P., Rasmussen, L., Houx, P., Rasmussen, H., & Canet, J. et al. (1998). Long-term postoperative cognitive dysfunction in the elderly: ISPOCD1 study. *The Lancet*, *351*(9106), 857-861. http://dx.doi.org/10.1016/s0140-6736(97)07382-0
7. Rabin, L., Barr, W., & Burton, L. (2005). Assessment practices of clinical neuropsychologists in the United States and Canada: A survey of INS, NAN, and APA Division 40 members. *Archives Of Clinical Neuropsychology*, *20*(1), 33-65. http://dx.doi.org/10.1016/j.acn.2004.02.005
8. Robertson, I., Manly, T., Andrade, J., Baddeley, B., & Yiend, J. (1997). `Oops!': Performance correlates of everyday attentional failures in traumatic brain injured and normal subjects. *Neuropsychologia*, *35*(6), 747-758. http://dx.doi.org/10.1016/s0028-3932(97)00015-8
9. Demakis, G. (2004). Frontal Lobe Damage and Tests of Executive Processing: A Meta-Analysis of the Category Test, Stroop Test, and Trail-Making Test. *Journal Of Clinical And Experimental Neuropsychology*, *26*(3), 441-450. http://dx.doi.org/10.1080/13803390490510149

**THE TRAIL MAKING TEST (TMT)**

|  | Method of Development | Developed by US Army as part of the AITB^1^; later used as an indicator of organic brain damage^1^ and incorporated into the HRNB^2^ |
| --- | --- | --- |
| Descriptive | Purpose | 2-part test used to detect neurological impairment^3^ |
|  | Content | Assesses attention, processing speed, mental flexibility^4^, memory, executive functioning^5^, and visuospatial tracking^6^  TMT A: 25 circles, numbered from 1 to 25, randomly distributed over the entire page^7^; measures visual search and motor speed skills^3^  TMT B: 25 circles, numbered 1 to 13 or lettered from A to L^7^; additionally measures mental flexibility^3^  CTMT: 5 parts, w/ 1-3 similar to TMT A and 4-5 similar to TMT B^6^ |
|  | Response -Options | Patient must connect circles in ascending order (alternating b/w numbers and letters for TMT B)^7^ |
|  | Recall Period | Not applicable |
|  | Endorsements | Available to public |
| Practical | To obtain | Available from Reitan Neuropsychology Labs: http://www.mcssl.com/store/  reitan-neuropsychologylaboratory/tests/trail-making-test-for-adults-item-18 |
|  | Method of Administration | Following 7-circle sample, patient must complete 25-circle task as fast as possible^4,7^ w/i 5-minute time limit w/o lifting pencil or making mistakes^4^ (though any mistakes can be quickly crossed out)^7^  Originally called for examiner to stop test after 3 errors, but now allows the examiner to point out errors so patient can correct them^8^ |
|  | Scoring and Interpretation | Most common system does not incorporate number of errors^7^; score for each part determined by # seconds required for completion^9,10^  Secondary: TMT B – A or B:A ratio (interference by mental flexibility)  Scores determined by comparing w/ normative for healthy populations^3^ |
|  | Respondent Burden | 5-10 minutes to complete^3^ |
|  | Admin Burden | No training required; must purchase forms, pencil/pen, and stopwatch^3^ |
|  | Translations | English, Arabic, Chinese, Hebrew |
| Critical appraisal value | Strengths, cautions, clinical and research applicability | Can be administered by itself or as part of a larger battery^3^  Easy to administer, quick and inexpensive^3^  Alternate forms for children aged 9-14 and adults >15^4^ |
|  |  | Significant difference in mean score b/w patients with ABI and healthy controls (p<0.001)^1^  TMT A can distinguish b/w frontal and non-frontal patients (d = -0.23; p < 0.05) but not b/w left and right frontal patients (d = -0.05; p ≥ 0.05)^11^; TMT B scores not observed to distinguish b/w frontal and non-frontal patients (t = 0.05-1.79; p = 0.10-1.00 or d = -0.16, p ≥ 0.05)^11,12^  TMT A weakly correlated w/ age (r = 0.225; p < 0.01) and NS correlated w/ education (r = 0.047; p > 0.01); TMT B weakly correlated w/ age and education (r = 0.278, -0.166; p < 0.01)^13^ |

ABI, acquired brain injury; AITB, Army Individual Test Battery; CTMT, Comprehensive Trail Making Test; TMT, Trail Making Test; US, United States

**References**

1. Reitan, R. (1955). The relation of the Trail Making Test to organic brain damage. *Journal Of Consulting Psychology*, *19*(5), 393-394. http://dx.doi.org/10.1037/h0044509
2. *In Depth Review of the Trail Making Test (TMT)*. (2017). *Stroke Engine*. Retrieved 1 August 2018, from http://www.strokengine.ca/indepth/tmt_indepth/
3. Bowie, C., & Harvey, P. (2006). Administration and interpretation of the Trail Making Test. *Nature Protocols*, *1*(5), 2277-2281. http://dx.doi.org/10.1038/nprot.2006.390
4. Wright, J. (2011). Trail Making Test. In: Encyclopedia of Clinical Neuropsychology. Springer International Publishing, pp.2537-2538.
5. Aslaksen, P., Ørbo, M., Elvestad, R., Schäfer, C., & Anke, A. (2013). Prediction of on-road driving ability after traumatic brain injury and stroke. *European Journal Of Neurology*, *20*(9), 1227-1233. http://dx.doi.org/10.1111/ene.12172
6. Smith, S., Servesco, A., Edwards, J., Rahban, R., Barazani, S., & Nowinski, L. et al. (2008). Exploring the Validity of the Comprehensive Trail Making Test. *The Clinical Neuropsychologist*, *22*(3), 507-518. http://dx.doi.org/10.1080/13854040701399269
7. Armitage, S. (1946). An analysis of certain psychological tests used for the evaluation of brain injury. *Psychological Monographs*, *60*(1), i-48. http://dx.doi.org/10.1037/h0093567
8. *Trail Making Test for Adults (Item #18)*. (2017). *Mcssl.com*. Retrieved 1 August 2017, from http://www.mcssl.com/store/reitan-neuropsychology-laboratory/tests/trail-making-test-for-adults-item-18
9. Lezak, M., Howieson, D., Bigler, E., & Tranel, D. (2012). *Neuropsychological assessment* (pp. 371-373). New York: Oxford University Press.
10. Tombaugh, T. (2004). Trail Making Test A and B: Normative data stratified by age and education. *Archives Of Clinical Neuropsychology*, *19*(2), 203-214. http://dx.doi.org/10.1016/s0887-6177(03)00039-8
11. Demakis, G. (2004). Frontal Lobe Damage and Tests of Executive Processing: A Meta-Analysis of the Category Test, Stroop Test, and Trail-Making Test. *Journal Of Clinical And Experimental Neuropsychology*, *26*(3), 441-450. http://dx.doi.org/10.1080/13803390490510149
12. Reitan, R., & Wolfson, D. (1995). Category test and trail making test as measures of frontal lobe functions. *The Clinical Neuropsychologist*, *9*(1), 50-56. http://dx.doi.org/10.1080/13854049508402057
13. Corrigan, J., & Hinkeldey, N. (1987). Relationships between Parts A and B of the Trail Making Test. *Journal Of Clinical Psychology*, *43*(4), 402-409. http://dx.doi.org/10.1002/1097-4679(198707)43:4<402::aid-jclp2270430411>3.0.co;2-e

**THE WECHSLER ADULT INTELLIGENCE SCALE (WAIS)**

| Descriptive | Method of Development | Developed from Wechsler–Bellevue Intelligence Scale (1939), which introduced splitting of FSIQ into VIQ and PIQ; developed as an intelligence test solely for adults (1955), and followed by WAIS-R (1981), WAIS-III (1997), and WAIS-IV (2008)^1,3^; WAIS-IV updated to improve clinical utility, developmental appropriateness, user friendliness, psychometric properties, and structural foundations^2-3^ |
| --- | --- | --- |
|  | Purpose | 15-item (10 core and 5 supplementary subtests)^1^ measure of cognitive ability in adults (ages 16 to 90)^2^ |
|  | Content | Assesses crystallized (VIQ) and fluid (PIQ) intelligence, as well as full mental ability (FSIQ)^3^  Four subscales including:  VCI – Info, Sim, and Vocab; Comp (supplemental)  PRI – BDT, MR, and VP (WAIS-IV); FW(WAIS-IV, ages 16-69 only) and PC (supplemental)  WMI – DG and Arith; LNS (supplemental, ages 16-69 only)  PSI – SS and DST; Cancel (WAIS-IV, supplemental, ages 16-69 only)^1,3^  Some subtests not included in WAIS-IV: OA, PA, DST Recall (DST-Incidental Learning), and DST Copy (DST-Copy)^1^ |
|  | Response -Options | Individual subtests are completed w/ paper and pencil or computer^2^ |
|  | Recall Period | Not applicable |
|  | Endorsements | Available to individuals w/ appropriate qualification^2^ |
| Practical | To obtain | WAIS-IV test kit available from Pearson Education: http://www.pearsonclinical.com/psychology/products/100000392/wechsler-adult-intelligence-scalefourth-edition-wais-iv.html^2^ |
|  | Method of Administration | Pencil and paper or web-based software ^2^ |
|  | Scoring and Interpretation | Scoring can be web-based or manual^2^  Composite scores:  FSIQ – from 10 core subtests^1^; mean = 100, SD = 15, range = 40 to 60^3^  VIQ and PIQ – WAIS-III and prior only^3^  Index Scores – VCI, PRI, WMI, PSI, GAI (VCI + PRI, optional)^1^; mean = 10, SD = 3^3^ |
|  | Respondent Burden | 60-90 minutes^2^ (Avg. 3-15 minutes for individual WAIS-III subtests)^4^ |
|  | Admin Burden | Requires training and Level C qualification, must purchase test kit and software^2^ |
|  | Translations | English, Chinese, Danish, Dutch, Finnish, French, German, Greek, Hebrew, Hungarian, Icelandic, Italian, Japanese, Korean, Lithuanian, Norwegian, Polish, Portuguese, Spanish, Swedish^5^ |
| Critical appraisal value | Strengths, cautions, clinical and research applicability | WAIS:  DST performance significantly slower for older age groups (t = 6.74; p < 0.01)^6^  WAIS-R:  BDT % Broken Configuration Errors weakly correlated w/ GCS (r = –0.26; p < 0.05); weak, NS correlations w/ age, education, and time post-injury (r = 0.05-0.10, –0.18; p > 0.05)^7^  Lower BDT performance associated with increasing age; higher education associated w/ less decline in performance ^8^  WAIS-III:  Education and presence of intracranial lesion were significant predictors of LNS (R^2^ = 0.04-0.12; p < 0.05) ^9^  Education was significant predictor of MR (R^2^ = 0.06; p < 0.05) ^9^  Education and presence of coma were significant predictors of SS(R^2^ = 0.04-0.21; p < 0.05) ^9^  Scores on tests involving information processing speed and perceptual organization seen to decrease with increasing age while verbal scores show minimal change ^10^ |

Arith, Arithmetic; BDT, Block Design Test; CAT, Category Test; CDN, Canadian; Comp, Comprehension; COWAT, Controlled Oral Word Association Test; DG, Digit Span; DST, Digit Symbol Test/Digit Symbol Coding/Coding; ESAT, Everyday Spatial Activities Test; FR, Free Recall; FSIQ, Full Scale Intelligence Quotient; FP, Family Pictures; FW, Figure Weights; GAI, General Ability Index; IQ, Intelligence Quotient; LM, Logical Memory; LNS, Letter Number Sequencing; MR, Matrix Reasoning; MRI, magnetic resonance imaging; NS, non-significant/non-significantly; OA, Object Assembly; PA, Picture Arrangement; PASAT, Paced Auditory Serial Addition Test; PC, Picture Completion; PIQ, Performance Intelligence Quotient; PPVT, Peabody Picture Vocabulary Test; PRI, Perceptual Reasoning Index; PSI, Processing Speed Index; RCFT, Rey Complex Figure Test and Recognition Trial; RMT, Road Map Test; SD, standard deviation; Sim, Similarities; SRA, Science Research Associates; SS, Symbol Search; STM, short-term memory; TBI, traumatic brain injury; TMT, Trail Making Test; VCI, Verbal Comprehension Index; VFD, Benton Visual Form Discrimination Test; VIQ, Verbal Intelligence Quotient; Vocab, Vocabulary; VP, Visual Puzzles; VPA, Verbal Paired Associates; VR, Visual Reproduction; WAIS (-R/III/IV), Wechsler Adult Intelligence Scale (-Revised/Third Edition/Fourth Edition); WBIS, Wechsler–Bellevue Intelligence Scale; WIAT-III, Wechsler Individual Achievement Test-Third Edition; WM, Working Memory; WMI, Working Memory Index, WMS-III, Wechsler Memory Scale-Third Edition; WRAT, Wide Range Achievement Test

**References**

1. *Introducing the WAIS-IV*. (2008). Retrieved 1 August 2017, from http://images.pearsonclinical.com/images/assets/WAIS-IV/WAISIV2_6_08.pdf
2. *Wechsler Adult Intelligence Scale-Fourth Edition*. (2017). *Pearsonclinical.com*. Retrieved 1 August 2018, from http://www.pearsonclinical.com/psychology/products/100000392/wechsler-adult-intelligence-scalefourth-edition-wais-iv.html
3. Saklofske, D., & Schoenberg, M. (2011). Wechsler Adult Intelligence Scale (All Versions). In: Encyclopedia of Clinical Neuropsychology. Springer International Publishing, pp.2675-2680.
4. Ryan, J., Lopez, S., & Werth, T. (1998). Administration Time Estimates for WAIS-III Subtests, Scales, and Short Forms in a Clinical Sample. *Journal Of Psychoeducational Assessment*, *16*(4), 315-323. http://dx.doi.org/10.1177/073428299801600403
5. *Content and Translations*. (2017). *Pearsonclinical.com*. Retrieved 1 August 2018, from https://www.pearsonclinical.com/pharma-licensing/content-and-translations.html
6. Salthouse, T. (1978). The Role of Memory in the Age Decline in Digit-Symbol Substitution Performance. *Journal Of Gerontology*, *33*(2), 232-238. http://dx.doi.org/10.1093/geronj/33.2.232
7. Wilde, M., Boake, C., & Sherer, M. (2000). Wechsler Adult Intelligence Scale-Revised Block Design Broken Configuration Errors in Nonpenetrating Traumatic Brain Injury. *Applied Neuropsychology*, *7*(4), 208-214. http://dx.doi.org/10.1207/s15324826an0704_2
8. Rönnlund, M., & Nilsson, L. (2006). Adult life-span patterns in WAIS-R Block Design performance: Cross-sectional versus longitudinal age gradients and relations to demographic factors. *Intelligence*, *34*(1), 63-78. http://dx.doi.org/10.1016/j.intell.2005.06.004
9. Donders, J., Tulsky, D., & Zhu, J. (2001). Criterion validity of new WAIS–III subtest scores after traumatic brain injury. *Journal Of The International Neuropsychological Society*, *7*(7), 892-898. http://dx.doi.org/10.1017/S1355617701246153
10. Ryan, J., Sattler, J., & Lopez, S. (2000). Age Effects on Wechsler Adult Intelligence Scale-III Subtests. *Archives Of Clinical Neuropsychology*, *15*(4), 311-317. http://dx.doi.org/10.1093/arclin/15.4.311

**THE WECHSLER MEMORY SCALE (WMS)**

| Descriptive | Method of Development | WMS developed in 1945 by adapting existing memory tests and combining with normative data; revisions include WMS-R (1987), WMS-III (1997), and WMS-IV (2009); WMS-IV updated to improve clinical utility, administration time, test items, and scoring rules; WMS-IV scores derived for Older Adult Battery (65-90) and Adult Battery (16-69)^1^ |
| --- | --- | --- |
|  | Purpose | To assess various domains of memory, including STM, LTM and WM^1^, in persons aged 16 to 90^2^ |
|  | Content | Standard WMS-IV subtests – LM I and II, VPA I and II, Designs I and II, VR I and II, SA, SS  Standard WMS-IV indexes – IM, DM, AM, VM, VWM  Subtests not included in standard WMS-IV battery – Logos I and II, Names I and II  Indexes not included in standard WMS-IV battery – VIM, VDM, AVM, AVIM, AVDM^3^  Subtests removed from WMS-IV (previous versions only) – I&O, SpS, MC, Faces, DG, FP, LN, WL^2^ |
|  | Response -Options | Individual subtests are completed w/ paper and pencil or computer^2^ |
|  | Recall Period | Not applicable |
|  | Endorsements | Available to individuals w/ appropriate qualification^2^ |
| Practical | To obtain | WMS-IV test kit available from Pearson Education: https://www.pearsonclinical.com/psychology/products/100000281/wechsler-memory-scale--fourth-edition-wms-iv.html^2^ |
|  | Method of Administration | Pencil and paper or web-based software^2^ |
|  | Scoring and Interpretation | Scoring can be web-based or manual^1^  Five composite scores: AM, VM, VWM, IM, DM  Contrast scores (WMS-IV) – scores on one subtest scaled based on performance on a related subtest^1-2^ |
|  | Respondent Burden | WMS-III – 30 to 35 min (primary subtests) + 15-20 min (supplemental subtests)^1^ |
|  | Admin Burden | Requires training and Level C qualification, must purchase test kit and software^2^ |
|  | Translations | English, Chinese, Czech, Danish, Dutch, Finnish, French, German, Italian, Japanese, Korean, Norwegian, Spanish, Swedish^4^ |
| Critical appraisal value | Strengths, cautions, clinical and research applicability | WAIS-R:  VIQ and PIQ were significant predictors of VeM score^5^  VIQ, PIQ, gender, # drugs, education, and seizure type were significant predictors of VM score^5^  VIQ, PIQ, and gender were significant predictors of GM score^5^ |

AL, Associate Learning; AM, Auditory Memory; AVDM, Auditory-Visual Delayed Memory; AVIM, Auditory-Visual Immediate Memory; AVM, Auditory-Visual Memory; DG, Digit Span; DM, Delayed Memory; DR, Delayed Recall; FP, Family Pictures; GM, General Memory; IM, Immediate Memory; IMIS, Inpatient Memory Impairment Scale; I&O, Information & Orientation; LM, Logical Memory; LN, Letter Number; LTM, long-term memory; MC, Mental Control; MQ, Memory Quotient; PIQ, Performance Intelligence Quotient; PTA, post-traumatic amnesia; SA, Spatial Addition; SpS, Spatial Span; SS, Symbol Span; STM, short-term memory; TMT, Trail Making Test; VDM, Visual Delayed Memory; VeM, Verbal Memory; VIM, Visual Immediate Memory; ViPA, Visual Paired Associates; VIQ, Verbal Intelligence Quotient; VM, Visual Memory; VPA, Verbal Paired Associates; VR, Visual Reproduction; VWM, Visual Working Memory; WL, Word List; WM, working memory; WAIS-I/R/III/IV; Wechsler Adult Intelligence Scale-First/Revised/Third/Fourth Edition; WMS-I/R/III/IV; Wechsler Memory Scale-First/Revised/Third/Fourth Edition

**References**

1. Chlebowski, C. (2011). Wechsler Memory Scale (All Versions). In: Encyclopedia of Clinical Neuropsychology. Springer International Publishing, pp.2688-2690.
2. *Wechsler Memory Scale-Fourth Edition*. (2017). *Pearsonclinical.com*. Retrieved 1 August 2017, from https://www.pearsonclinical.com/psychology/products/100000281/wechsler-memory-scale--fourth-edition-wms-iv.html#tab-details
3. *WMS-IV Flexible Approach. Retrieved from https://images.pearsonclinical.com/images/Products/WMS-IV/slide2.pdf*
4. *Content and Translations*. (2017). *Pearsonclinical.com*. Retrieved 1 August 2017, from https://www.pearsonclinical.com/pharma-licensing/content-and-translations.html
5. Moore, P., & Baker, G. (1997). Psychometric properties and factor structure of the wechsler memory scale-revised in a sample of persons with intractable epilepsy. *Journal Of Clinical And Experimental Neuropsychology*, *19*(6), 897-905. http://dx.doi.org/10.1080/01688639708403770
